# Review on Gallium in Coal and Coal Waste Materials: Exploring Strategies for Hydrometallurgical Metal Recovery

**DOI:** 10.3390/molecules29245919

**Published:** 2024-12-15

**Authors:** Ewa Rudnik

**Affiliations:** Faculty of Non–Ferrous Metals, AGH University of Krakow, Al. Mickiewicza 30, 30-059 Krakow, Poland; erudnik@agh.edu.pl

**Keywords:** gallium, coal, coal gangue, fly ash, bottom ash, occurrence mode, leaching, separation

## Abstract

Gallium, a critical and strategic material for advanced technologies, is anomalously enriched in certain coal deposits and coal by-products. Recovering gallium from solid residues generated during coal production and utilization can yield economic benefits and positive environmental gains through more efficient waste processing. This systematic literature review focuses on gallium concentrations in coal and its combustion or gasification by-products, modes of occurrence, gallium-hosting phases, and hydrometallurgical recovery methods, including pretreatment procedures that facilitate metal release from inert aluminosilicate minerals. Coal gangue, and especially fly ashes from coal combustion and gasification, are particularly promising due to their higher gallium content and recovery rates, which can exceed 90% under optimal conditions. However, the low concentrations of gallium and the high levels of impurities in the leachates require innovative and selective separation techniques, primarily involving ion exchange and adsorption. The scientific literature review revealed that coal, bottom ash, and coarse slag have not yet been evaluated for gallium recovery, even though the wastes can contain higher gallium levels than the original material.

## 1. Introduction

Gallium is a multifunctional element with unique and intriguing properties, making it a key component in various applications. It is a soft, silvery-white post-transition metal with amphoteric chemical properties [1]. Solid gallium is magnetic (diamagnetic) and a good conductor of both electricity (7.1 × 10^6^ S/m) and heat (29 W/(m∙K)). The element exhibits low vapor pressure, even at high temperatures (from 6.3 × 10^−9^ bar at 727 °C to 1 bar at 2204 °C). Gallium has a low melting point (29.76 °C) and a high boiling point (2204 °C), resulting in an exceptionally wide temperature range in its liquid state. The density of liquid gallium is 3.2% higher than that of its solid form, which is an unusual and anomalous property for a metal. Due to its tendency to resist crystallization, gallium can be easily undercooled, remaining liquid even below its melting point, up to −123 °C. However, once crystallization nuclei form, it solidifies expanding by 3.1%. At room temperature, gallium has an orthorhombic crystal structure (α–Ga), but it can form seven different crystal lattices depending on crystallization conditions or under pressure [2].

Unlike most metals, gallium is often called a “metallic molecular crystal” [3] or “molecular metal” [4], i.e., that is molecular and metallic at the same time. Gallium atoms can form covalently bonded dimers Ga_2_ connected by metallic bonding, primarily occurring in the plane perpendicular to their average alignment. A recent study [5] has shown that covalency becomes more important in liquid gallium at higher temperatures, explaining the decrease in resistivity of the metal upon melting and its subsequent anomalously nonlinear increase with temperature. These unique properties, combined with low vapor pressure, low reactivity, and low toxicity, distinguish gallium from other liquid metals and alloys (e.g., mercury, cesium, sodium–potassium alloy), making it ideal for advanced applications such as soft robots, wearable electronic devices, and catalyst for energy transformation and 2D material synthesis [6,7].

Gallium was first recognized as a strategic and critical metal in the 1940s when it became an essential component of a stable gallium–plutonium alloy developed for the Manhattan Project (USA) to produce an atomic bomb [8]. Today, gallium remains a critical mineral for many countries, including the USA [9], Canada [10], the European Union [11], the United Kingdom [12], Japan [13], India [14], China [15], due to its significant economic importance across key sectors such as electronics, automotive, aerospace, defense, healthcare, and environmental technologies [8,10,12,16]. The semiconducting and optoelectronic properties of gallium compounds, such as gallium arsenide GaAs, gallium nitride GaN, gallium phosphide GaP and copper indium gallium selenide CIGS, make them essential materials for high-performance electronics and photovoltaics. The main applications of gallium-based materials include high-frequency integrated circuits (chipsets) in mobile and satellite communication, 5G wireless base stations, laser diodes in fiber-based communication systems for high-speed data transmission, energy efficient and durable LED lighting and displays, infrared/ultraviolet lasers, advanced diagnostics and medical devices, highly effective thin-film solar cells and production of NdFeB magnets for efficient energy conversion in zero-emission vehicles and wind turbines as well as essential for sensors in avionics, space, and military systems and automotive applications [1,17,18,19].

**Figure 1:**
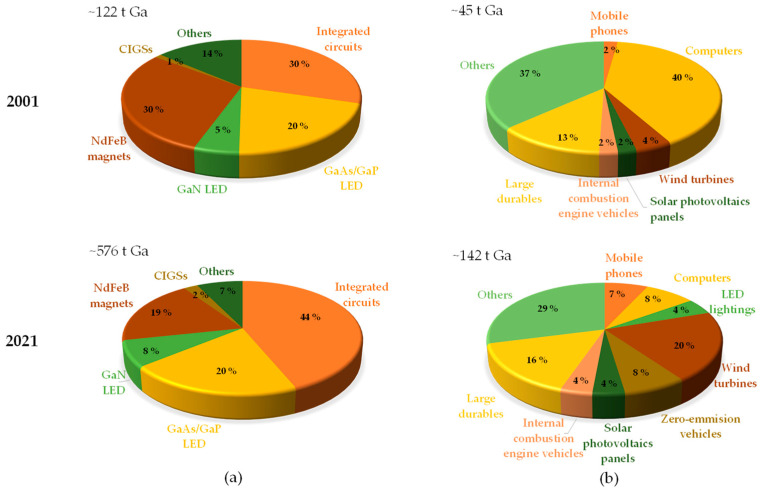
Structure of gallium consumption in (**a**) semi-products and (**b**) final products based on global gallium flows in 2001 and 2021 taken from [15].

The global production of gallium-containing semi-products (i.e., integrated circuits, LEDs, magnets) is driven by the end-use products (e.g., mobile phones, solar photovoltaic panels, electric vehicles), the structure of which has evolved over the last two decades [15]. Figure 1 illustrates the key applications of gallium based on the tonnage consumed in the production of semi-products and final products in 2001 and 2021. All of these applications are crucial for future technologies required for the green transition, digitalization, and defense, making gallium a strategic raw material with significant supply concerns due to its high import dependence and limited availability [15,19,20,21,22,23].

**Figure 2:**
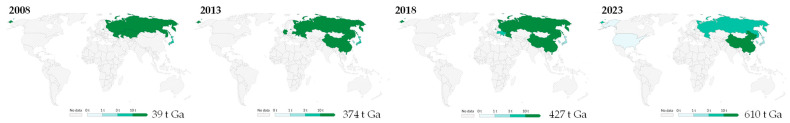
Global changes in gallium primary production over the last fifteen years: gallium production levels (in tons) by country in 2008, 2013, 2018 and 2023 [25].

Rising demand for gallium in key industries has heightened the need to secure a stable supply of the mineral. The gallium market is relatively small in terms of quantity, but it is simultaneously a high-supply-risk market, referring to the high concentration of gallium-supplying countries [22]. Mitigating supply chain risks has become especially important since China, the world’s leading producer of gallium (Figure 2), imposed export restrictions on this resource starting 1 August 2023 [21]. Consequently, this results in a necessity for restructuring the global gallium supply chain, a search for new resources and opportunities to launch production, changes in international trade flows and pricing mechanisms, as well as fluctuations in metal prices [8,15,21,22,23]. Notably, gallium is not traded on any metals exchange, nor are there markets for establishing an official price. Instead, gallium prices are determined through long-term bilateral negotiations between suppliers and buyers [22]. Over the past few years, the price of gallium (min. 99.995%) has shown a trend of increasing volatility, reaching almost USD 920 per kilogram in November 2024, over double the prices in May–July 2023, and near to the June–July 2022 record prices [24].

Gallium is a dispersed element in the continental crust and does not form its own ore deposits [26]. Rare gallium minerals are gallite CuGaS_2_, söhngeite Ga(OH)_3_ and krieselite (Al,Ga)_2_(GeO_4_)(OH)_2_ occurring in some African regions (DR Congo, Namibia) [1]. Instead, gallium exists in trace amounts within minerals and rocks, substituting atoms of elements with similar sizes and charges, such as zinc or aluminum. As a result, gallium is found as an accompanying element in zinc and zinc–lead sulfide ores (in sphalerite ZnS) [27,28,29] and in aluminum-bearing minerals like diaspore γ–AlOOH, boehmite α–AlOOH and gibbsite Al(OH)_3_, which are components of bauxite deposits [30,31]. Table 1 shows the concentration ranges of gallium in these natural sources.

About 90–93% of gallium comes from the aluminum industry as a by-product of the smelting process of bauxite (the Bayer process), while the remaining sources from zinc concentrates (i.e., extracted from zinc smelting slag) [15,34,35]. As China is a global leader in aluminum production, it also dominated gallium supply, with shares of about 58% and 98.4% in 2023, respectively [25]. Although bauxite deposits can contain over one million tons of gallium, and rich zinc ore reserves (approximately 1.9 billion tons) indicate their significance as global sources of gallium [26], coal has been identified as a third natural source.

Over the past twenty years, reports have noted anomalous enrichment of gallium in some coals [36] and it is estimated that coal can contain about 10 million tons of gallium as a potential reserve [26]. This also converts to the presence of gallium in coal gangue and its concentration in ashes produced during coal and coal gangue combustion [37]. Although the very idea of extracting gallium from coal and coal ash is not new [38], today’s challenges and technological opportunities make the process feasible, especially since the detected levels of gallium have sparked interest as prospective economic reservoirs of the element. Therefore, this paper aims to provide an overview of the opportunity for recovering gallium from coal, specifically from coal gangue, coal ashes and slag as unconventional secondary sources. This paper particularly focuses on hydrometallurgical methods of recovery to highlight advancements, outline the factors influencing process effectiveness, and identify the limitations and challenges in this area. Although coal waste materials are not currently utilized as a source of valuable elements, numerous research studies highlight their potential, and this may become a viable option in the future. It is particularly relevant given the global industry’s focus on resource diversification and the economic and environmental benefits of waste utilization. This approach is additionally worthwhile as the recovery of gallium from recyclable electronic end-of-life products and scrap is still in the very early stages of development [39,40,41].

## 2. Gallium in Coal

### 2.1. Gallium-Bearing Coal Seams

Gallium was detected in coals long before it became a material of industrial importance [38]. Average concentrations of gallium in coal worldwide are generally in the range of 1–10 ppm [26,36], though some deposits may locally contain as much as 20–30 ppm or even much more (Table 2). The mean gallium content in samples of mined coal from over fifty countries, collected in the World Coal Quality Inventory database (U.S. Geological Survey) [42], can be estimated at 8.2 ± 6.9 ppm (on a dry, whole-coal basis).

Gallium is not an element regularly analyzed in coal, so it can often be overlooked. However, the discovery of gallium-rich coal seams in China in 2006 [43] stimulated geological exploration aimed at identifying deposits enriched in this element. Anomalously enriched, super-thick coal beds (containing 12–76 ppm Ga, with a mean of 45–52 ppm) were found in the Heidaigou Mine of the Jungar Coalfield in the Ordos Basin, where the main minable benches account for about 82% of the total coal bed thickness. It was estimated that these reserves could contain between 63,000 and 860,000 tons of gallium. Further investigations have identified other gallium-rich coal deposits [36], for example, in the Huangling Mine in the Huanglong Coalfield (21–249 ppm Ga) [36], the Dongpo Mine in the Weibei Coalfield (26–59 ppm Ga, with a mean of 31.6 ppm) [44], and the Pingshuo mine district in the Ningwu Coalfield (primarily averaging 20–40 ppm Ga) [45]. In the latter location, gallium reserves are estimated to be approximately 150,200 tons. Recent data [46] indicate gallium concentrations of 10–747 ppm (mean of 157 ppm) in Cambrian stone coal-bearing seams within the South Qinling Orogenic Belt in central China, corresponding to estimated reserves of around 100,600 tons of gallium. Numerous coal deposits in China exhibit exceptionally high gallium levels [36,47], exceeding the domestic average, estimated at 6.6–9.0 ppm [48,49]. Notably, average gallium concentrations as high as 15.5 ppm were found in Permo-Carboniferous coals, with seams very often containing more than 30 ppm Ga [50]. Nevertheless, coal seams locally enriched in gallium have no commercial value in most Chinese coalfields. Qin et al. [36] have shown that among about 20 gallium-enriched coal areas, only a few have potential economic importance: the Heidaigou Mine (12–76 ppm Ga) in the Jungar Coalfield, the Pingshuo Mine (8.2–68 ppm Ga) in the Ningwu Coalfield, and the Gequan Mine (3.8–46 ppm Ga) in the Xingtai Coalfield. It should be noted that 30 ppm is recommended as the cut-off grade for the commercial recovery of gallium from coal in China [36,43].

**Table 2:** Gallium concentration in world’s coals.

Region	Concentration Range (Mean), ppm	Ref.
World	(5.8)	[50]
Brown coal	5.5 ± 0.3	[50]
Hard coal	6.0 ± 0.2	[50]
Australia	–	
Bowen Basin	7–21	[51]
Bulgaria	–	
Pernik Basin	(26.5)	[52]
China	(6.5–6.8)	[48]
Guizhou Province	2.5–30.7 (10.7)	[53]
Heshan Coalfield	12.3–38.2 (22.6)	[54]
Huanglong Coalfield	11.5–249 (89)	[36]
Jungar Coalfield	3.5–76 (18–26)	[43,55,56]
Ningwu Coalfield	11.1–33.8 (20.1)	[57]
Pingshuo Coalfield	11.9–24	[49]
Songshan Coalfield	11.8–17	[49]
Xingtai Coalfield	10.3–23.9	[49]
South Qinling Orogenic Belt	10–747 (157)	[47]
Egypt	1–34 (6.5)	[58]
Indonesia		
Barito Basin	0.6–15.2 (5.4)	[59]
Iran	0.9–37.3 (10.9 ± 9.0)	[60]
Kazakhstan	0.1–5	[61]
Karaganda Basin; k7 seam	8.5–10.2 (9.6)	[62]
Nigeria	1.8–22.2 (5.2)	[58]
Poland	–	
Brown coal; Katowice region	0.6–7.0 (3.5 ± 3.2)	[63]
Hard coal; Bełchatów region	0.8–17.0 (8.2 ± 6.0)	[63]
Lublin Basin	0.6–5.2 (3.0)	[64]
Russia	–	
Kuznetsk Basin	0.1–4.1	[65,66]
Pavlovsk Coalfield, Spetsugli	8.1–27.3 (14.5)	[67]
Sakhalin Basin	2.0–9.8 (4.4 ± 0.3)	[68]
South Africa	8.2–16.9 (11.5)	[58]
Spain	–	
Power plant (unspecified)	(10)	[69]
Tanzania	9.3–14.7 (13.0)	[58]
Turkey	0.85–20 (5.8)	[70]
Cayirhan Coalfield	(6.6 ± 0.2)	[71]
Kangal Coalfield	5.0–8.3 (6.8)	[72]
Sorgun Basin	(9.6)	[73]
United Kingdom	0.6–7.5	[74]
Parkgate coal bed	(3.3)	[75]
USA	0.04–41 (5.1–5.2)	[76,77]
Appalachian Basins	5.4–6.9	[78]
Danville Seams, Indiana	1.7–8.9 (5.1)	[78]
Gulf Coast Basin	(6.9)	[78]
Illinois Basin	0.8–11 (4.8)	[78,79]
Springfield Seams, Indiana	1.4–12.3 (3.4)	[78]
Zambia	1.7–14.7 (9.9)	[58]

Other countries have also commenced more attention to the gallium content of their coal deposits [51,58,60,64,65,76,80]; however, data on this topic are relatively scarce. These studies usually report the element’s concentration in a limited number of samples, but they are not as extensive or systematically planned as in the case of Chinese coal deposits.

Lin et al. [77] conducted an analysis of promising U.S. coal samples for gallium recovery based on data from the coal quality database USGS. Considering the mean gallium concentration of 5.1 ppm, they found that approximately 5.8% of coal samples met the established threshold of 100 ppm gallium in coal ash. It was noted that bituminous coals from the Central Appalachian region, specifically in Kentucky, are likely to be a significant source of the element. In this area, gallium concentrations in Gray Hawk coal have been observed at levels of 2–9 ppm [81]. An average value of 5.4 ppm Ga was reported in the Central Appalachian Coal Basin, which is lower than in the Northern and Southern Appalachian Basins, with values of 6.9 ppm and 6.2 ppm, respectively [78].

An analysis of statistical data on gallium occurrence in African coals was performed by Chelgani [58]. He evaluated nearly 140 samples from six countries (Botswana, Egypt, Nigeria, South Africa, Tanzania, and Zambia) using data from the World Coal Quality Inventory. The mean gallium concentration was higher than the global average, with Tanzanian coals exhibiting the highest average contents (primarily in the range of 11–14 ppm). Interestingly, Egyptian coals showed a wide distribution of gallium concentrations (mainly in the range of 5–17 ppm), with values as high as 34 ppm observed rarely.

Relatively high gallium contents have also been reported for some coals in the Russian Far East, averaging 14 ppm in germanium-bearing seams of the Spetsugli deposits in the Pavlovsk Coalfield [67]. However, analyses of samples from various coal areas in Sakhalin [68] and the Siberian Kuznetsk Basin [66] revealed gallium concentrations of around 4.2 ppm, which is below the global average. Somewhat higher concentrations (5.7–6.2 ppm Ga) were identified in a seam of the Izykh Coalfield (Minusinsk Basin), where abnormally high gallium concentrations of 15.7–72.2 ppm were found in coal-bearing rocks and rock parting [82].

**Figure 3:**
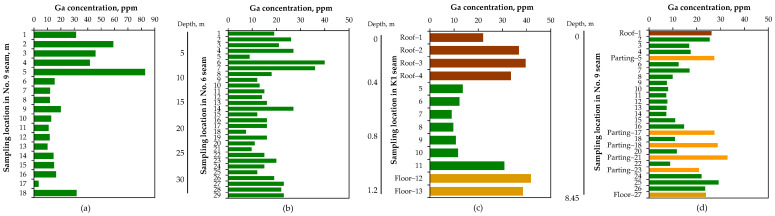
Vertical distribution of gallium in Chinese coal seams: (**a**) No. 9, Anjialing Mine, Ningwu Coalfield [84], (**b**) No. 6, Haerwusu Mine, Jungar Coalfield [88], (**c**) K1, Daping Mine, Yudongnan Coalfield [89], (**d**) No. 9, Antaibao Surface Mine, Ningwu Coalfield [90].

It should be noted that the distribution of gallium is heterogeneous, both in horizontal planes and vertical profiles within the same coal seams [45,49,55,83], due to factors such as coal formation conditions, regional tectonic activity, volcanic processes, and related phenomena [48,84,85,86]. In the vertical sections of coal seams, gallium content varies, with the topmost and basal parts of the seam often containing different average concentrations than the middle sections (Figure 3). For example, Mastalerz and Drobniak [78] compared gallium distribution in two southwestern Indiana seams at several mine locations (USA). They found that the lowest gallium concentrations were consistently in the middle portions of Springfield Coal at all four locations investigated. In contrast, the gallium distribution in the Danville Coal showed asymmetric changes: in the Farmersburg Mine, the lowest concentrations were observed in the topmost benches, with an increasing trend toward the bottom of the seam, whereas in the Air Quality Mine, the opposite pattern was identified, with the lowest concentrations at the bottom and the highest at the topmost part. Notably, gallium concentrations in the associated clastic rocks were high. In Springfield Coal (mean 3.4 ppm Ga in coal), they ranged from 16–43 ppm (mean 26.6 ppm) in the roof part to 22–49 ppm (mean 33.2 ppm) in the floor, with 20–45 ppm (mean 34.9 ppm) in the parting layer.

Shao et al. [87] examined the vertical distribution of gallium in coals from the Qugugou Mine in the Muli Coalfield, China. They found that the degree of gallium enrichment varies significantly depending on coal-forming periods, with element concentrations in coal areas near the roof, floor, and partings being relatively higher than in other regions. This was attributed to gallium migration due to groundwater leaching during the coal formation. Notably, gallium concentrations in the carbonaceous mudstones overlying the coals showed the highest levels, exceeding 35 ppm. The average concentrations across three mines in the Muli Coalfield range from 30 ppm to 37 ppm Ga.

The degree of gallium enrichment in coal is often evaluated using the concentration coefficient CC, defined as the ratio of an element’s concentration in a sample to its average concentration in world (or domestic) coals. Based on CC values, coals are classified into six groups: depleted (0 < CC < 0.5), normal (0.5 < CC < 2), slightly enriched (2 < CC < 5), enriched (5 < CC < 10), significantly enriched (10 < CC < 100), and unusually enriched (CC > 100) in gallium [48,54,57,84,90]. Accordingly, Chinese gallium-rich coal deposits generally fall into the enriched category, with fewer seams classified as significantly enriched. For instance, coal from the Huangling Mine, with a mean gallium concentration of 89 ppm, has a concentration coefficient of 15.9.

### 2.2. Modes of Occurrence

Elements in coal can be associated with inorganic substances or organic compounds, either chemically or physically bound. They may also be dissolved in pore waters or oils within the coal or adsorbed onto the surface of organic matter. Various methods are employed to investigate the modes of element occurrence in coal, including statistical analyses, density separation, selective leaching, X-ray diffraction and a range of microscopic, spectroscopic, and spectrometric techniques. These methods provide structural information as well as elemental and isotopic composition [91,92,93,94,95]. However, due to variations in spatial resolution and detection limits, careful selection of analytical methods is crucial. This selection should consider the sensitivity required to detect trace amounts of gallium in coal, along with proper sample preparation and analysis protocols.

Gallium in coals exhibits a complex nature of occurrence, as its association with multi-mineral and/or organic materials has been observed in coals from different geological periods and with varying gallium concentrations [36,46,49,96,97]. Gallium often correlates positively with ash yield [43,53,55,60,73,82], suggesting that its occurrence is predominantly associated with inorganic matter, primarily aluminum and/or aluminosilicate minerals due to isomorphic aluminum substitution [43,47,55,93,96,98], as well as sulfide minerals such as pyrite and sphalerite [46,55,62]. Depletion of ash suggests an association of the element with organic matter [55]. As a result, conclusions on gallium speciation are predominantly based on statistical correlations with other elements/oxides (e.g., Al, Si, S) [46,53,55,57,62,89,99] or correlation coefficients with ash yield [53,54,55,100]. Sequential leaching methods, which determine the distribution of gallium among silicate (or residual), organic and/or sulfide (oxidizable), carbonate (or acid-soluble), iron–manganese oxide (or reducible), ion-exchangeable, and water-soluble forms, are used less frequently [45,101,102], despite their ability to quantify gallium distribution among individual fractions. Advanced multiscale in situ investigation techniques (e.g., Backscattered Electron-Energy-Dispersive X-ray Spectroscopy (BSE–EDS), Electron Microprobe Analysis (EMPA) mapping, Laser Ablation Inductively Coupled Plasma Mass Spectrometry (LA–ICP–MS)) provide more detailed insights, identifying modes of occurrence of trace elements, such as gallium enrichment within gelatinous components of macerals in the organic matter of coal [95,97].

Table 3 presents the modes of gallium occurrence in selected coals. Studies have shown that boehmite and kaolinite are the primary sources of gallium in the Jungar coal [43,55,95,98]. Coal deposits with high concentrations of this element and aluminum are feasible, given that the association between coals and bauxites has been identified in several regions [45,57,98,103].

The speciation of gallium in coals provides insight not only into the sources of the element during coal bed formation and geochemical processes but is also crucial for understanding its behavior during coal combustion, beneficiation, conversion, and technological applications, enabling assessment of its potential as a valuable resource.

### 2.3. Gallium Recovery

Although some coal deposits contain unusually high concentrations of gallium, these are not exploited as a source of the element due to their dispersion within coal-bearing strata. Recently, Zhang et al. [83] discussed a cooperative exploration model from both theoretical and economic perspectives, using the Heidaigou coal–gallium deposit in China as an example. This model addresses the uneven distribution of gallium within the seam, exploration techniques for both coal and gallium, exploration methodologies, resource estimation, and other factors necessary for developing economically efficient, integrated resource utilization.

There are no research studies on direct hydrometallurgical recovery of gallium from coal. However, Zou et al. [104] reported an extraction procedure from gallium-enriched tuff in a coal-bearing strata-hosted rare metal deposit at the Zhongliangshan mine, China. The sample consisted mainly of kaolinite, pyrite, anatase, and jarosite. The following steps were applied: (i) alkaline sintering at 860 °C for 30 min with anhydrous sodium carbonate, (ii) water immersion at 90 °C for 120 min, and (iii) acid leaching with 4 M HCl at 40 °C, at a solid-to-liquid ratio of 20:1. This resulted in a 93.4% recovery of gallium. Interestingly, water leaching alone achieved a recovery rate of 67.3%.

The assessment of the economic feasibility of gallium recovery from coal is based on cut-off grade and deposit size. The cut-off for gallium in Chinese coal is 30 ppm [43], while the industrial grade is 50 ppm and a coal seam thickness of 5 m [36,48,83]. Wang et al. [53] conducted a correlation analysis combining data from coals and coal ashes worldwide and proposed a cut-off of 60 ppm for gallium, which corresponds to a gallium content of 100 ppm in ash. The evaluation of gallium-bearing coals from other regions of the world generally relies on cut-off values developed for Chinese deposits, although 20 ppm was a concentration recommended for further assessment for Russian coal [65,80].

## 3. Gallium in Coal Combustion Products

### 3.1. Gallium in Ashes and Slag

**Figure 4:**
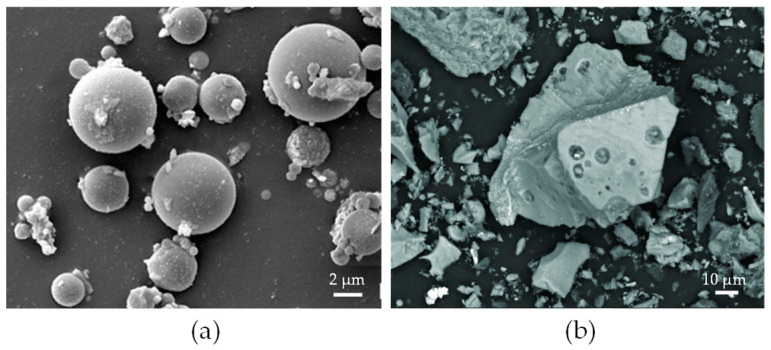
SEM images of coal fly ash (**a**) [107] and bottom ash (**b**) [108].

Coal combustion in coal-fired power plants generates various waste materials collectively referred to as coal ash [105], primarily involving fly ash, bottom ash, and boiler slag. Among these, coal fly ash accounts for the majority, covering approximately 40–90% of the total combustion residues. It is a fine (10–200 μm), powdery material (Figure 4a) that is captured from flue gas and collected using electrostatic or mechanical precipitation systems. It is estimated that for every four tons of coal burned, one ton of fly ash is produced, amounting to approximately 600–800 million tons generated annually worldwide [106].

Bottom ash, on the other hand, makes up 10–20% of the total coal ash waste [109]. It consists of agglomerated particles that are too large to be carried into the flue gases, causing them to accumulate at the bottom of the coal furnace. This material is granular and highly porous (Figure 4b), with particle sizes reaching up to several dozen millimeters. The global annual production of coal bottom ash is estimated at 780 million tons, with 66% originating from Asian countries, followed by contributions from Europe and the USA [110]. Although both types of ash can be beneficially reused, their large percentages are still stored in landfills and ash ponds, leading to significant environmental concerns [105,109]. However, given gallium’s typical inorganic affinity in coal and its concentration in ashes, the latter appears to be a highly suitable feedstock for gallium recovery.

During coal combustion, gallium accumulates in both fly and bottom ashes, although with a lesser tendency in the latter [111,112,113,114,115]. It is estimated that gallium enrichment in fly ash is approximately 1.6 to 1.8 times higher than in bottom ash due to its volatility behavior [111,113]. Zhou et al. [113] stated that during coal combustion, 64% of gallium accumulates in fly ash, approximately 19% in bottom ash, and the remaining 17% is carried away with the flue gas. The gallium content in ashes is influenced by its concentration in the feed coal and is up to ten times higher than in the raw material. The average concentration of gallium in coal fly ash worldwide is 33 ppm [50], with somewhat higher levels found in ashes derived from hard (bituminous) coals compared to brown (lignite) coals. In some cases, industrially collected materials have shown gallium concentrations, exceeding the cut-off value of 100 ppm [53] and indicating their economic potential. The highest gallium content was reported to be 1.5% in a single sample of coal fly ash collected from one of the UK industrial installations [38]. Table 4 shows gallium concentrations in coal and coal ashes from different regions of the world.

A third by-product of coal combustion is boiler slag. It is generated exclusively in specialized boilers designed to keep bottom ash in a molten state until it is removed. The resulting slag consists of granular, hard, incombustible black glassy particles, typically up to a few millimeters in size [105]. Due to its utility and limited production, nearly all of the boiler slag produced is repurposed. Data on gallium concentrations in boiler slag are limited. Xu et al. [126] reported that the gallium content in boiler slag (23 ppm) is similar to that found in bottom ash (20 ppm) but is about half of the concentration in fly ash (40 ppm).

### 3.2. Modes of Gallium Occurrence

**Figure 5:**
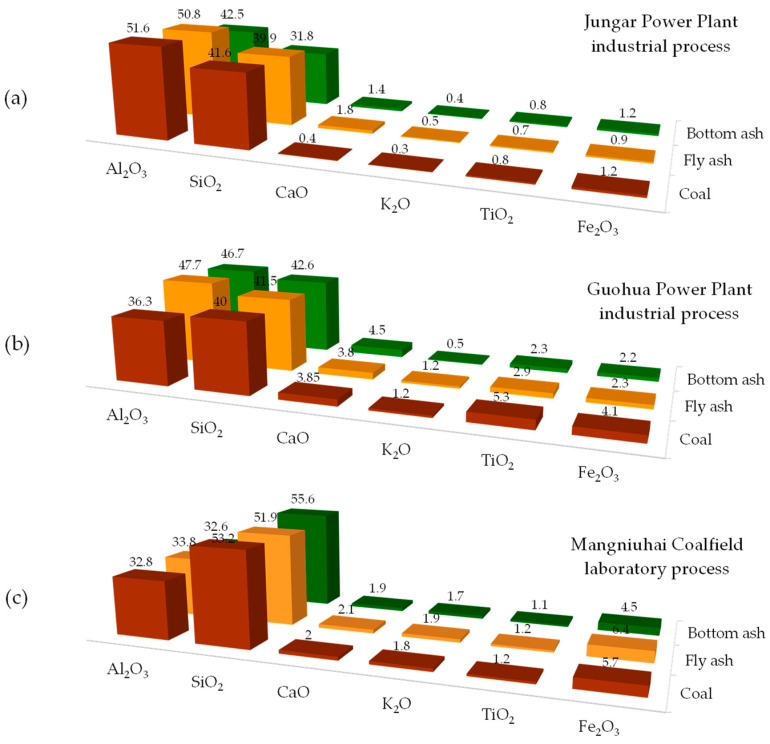
Main components in feed coal from different seams and ashes produced in industrial (**a**) [116], (**b**) [114] and laboratory conditions (**c**) [113].

Coal fly and bottom ashes as well as boiler slag are primarily composed of silicate and aluminosilicate glass, along with minerals and carbonaceous combustion fragments, with their proportions varying depending on the coal type and combustion conditions [37,113,114,115,116,118,123,124,125,126,127,128,129,130,131,132,133,134,135,136,137,138,139,140,141,142,143,144,145,146,147,148]. The mineral components present in variable amounts include quartz, mullite, lime, hematite, magnetite, and gypsum, among others. Figure 5 compares the compositions of feed coals with coal combustion fly and bottom ashes that have been either industrially collected or produced in laboratory settings.

Chen et al. [146] showed that the mineral proportions in coal fly ash depend on the type of combustion, i.e., conducted in a pulverized coal boiler PC (at 1000–1500 °C) or a circulating fluidized bed boiler CFB (at 800–900 °C with calcite addition for controlling the emission of SO_2_). Thus, PC fly ash mainly consisted of quartz and mullite, with calcium oxide present in an amorphous phase. In contrast, quartz, anhydrite, calcite, portlandite, and albite, but not mullite, with calcium oxide predominantly in a crystalline phase, were found in CFB fly ash. The glass phase content slightly differs between the two types of fly ash, accounting for approximately 43% in PC fly ash [148] and 50% in CFB fly ash [127].

Feng et al. [114] analyzed the transformations of minerals during coal combustion at different temperatures (600–1200 °C). The feed coal consisted primarily of kaolinite (68%), boehmite (18%), quartz (8%), and calcite (4%). These minerals were transformed mainly into the glass phase (62% and 57%), mullite (31% and 34%), and corundum (4% and 3%) in fly ash and bottom ash, industrial by-products. Laboratory investigations under simulated combustion conditions showed that the proportion of specific mineralogical components in the ash depends on the combustion temperature. The fraction of the glass phase gradually decreased from nearly 92% at 600 °C to 44% at 1200 °C. Mullite was detected only at higher combustion temperatures (21% at 1000 °C and 39% at 1200 °C), while the percentage of corundum slightly increased from 0.9% at 600 °C and 800 °C to 2.7% at 1200 °C.

During coal combustion, gallium is distributed between different phases. Frandsen et al. [128] modeled the thermodynamic behavior of gallium during coal combustion. They concluded that the main stable species of gallium before a temperature of about 1100 °C is Ga_2_O_3_ (crystal), while at higher temperatures the equilibrium distribution of gallium can be controlled by different proportions of GaCl, GaO, Ga_2_O, Ga and/or Ga_2_S in the gaseous states.

**Figure 6:**
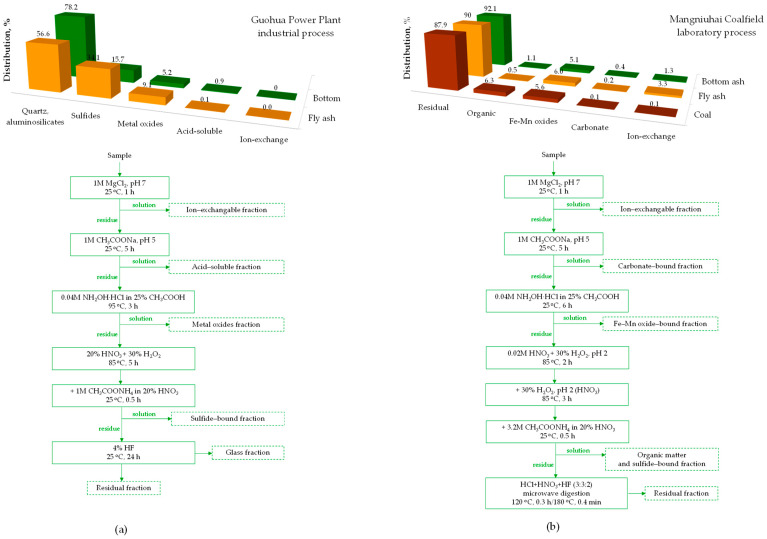
Percentage distribution of gallium in feed coal and ashes produced in industrial (**a**) [114] and laboratory (**b**) [113] conditions with sequential leaching procedure used.

Recent studies have provided a more detailed understanding of the modes of gallium occurrence in both fly and bottom ashes, using sequential leaching procedures, thermodynamic calculations, and compositional characterization [113,114]. Feng et al. [114] analyzed ash samples from a Chinese power plant (Figure 6a). They found that quartz and aluminosilicates were the main gallium-hosting minerals, with a higher fraction in bottom ash (78%) compared to fly ash (57%). In turn, the sulfide-bonded and metal oxide fractions showed a greater share of gallium in fly ash than in bottom ash. The remaining ash fractions (i.e., acid-soluble and ion-exchangeable) exhibited similar gallium distributions at trace levels of 0–0.9%. Detailed analysis of coal ash produced under laboratory conditions revealed that the distribution of gallium changes with combustion temperature (600–1200 °C). At 600 °C, over 80% of gallium migrated from kaolinite and boehmite into the glass phase. However, at higher temperatures, it primarily transferred into mullite and quartz. Consequently, more than 95% of gallium accumulated in the quartz–aluminosilicate fraction in ashes produced at 1000–1200 °C, compared to only 50–60% in ashes formed at lower temperatures. This pattern aligns with the gallium speciation observed in industrial ashes, where mullite and quartz were identified as dominant modes of occurrence. At combustion temperatures of 800–1000 °C, solid gallium sulfide Ga_4_S_5_ transformed into gaseous Ga_2_S, leading to a significant reduction in the sulfide-bonded gallium fraction from 35% in ash produced at 800 °C to below 4% in ashes formed at 1000 °C or higher.

These findings were consistent with the data for coal fly and bottom ashes produced under simulated laboratory conditions at 1000 °C [113]. Although a somewhat different sequential leaching procedure was applied (Figure 6b), approximately 90% of gallium was found to accumulate in the residual fraction, similar to its distribution in the original coal. The distribution of gallium in iron–manganese oxides and carbonates was comparable across all three materials. Notably, there was an increased presence of gallium in the ion-exchangeable form in ashes compared to the coal sample. In contrast, there was a reduced association of gallium with organic matter, which is expected given the transformation that organic material undergoes during coal combustion.

Xu et al. [126] conducted a more detailed analysis of fly ash from the Chongqing Power Plant, using a modified sequential leaching procedure described by Feng et al. [114]. This method involved separating the glass phase from the mullite–quartz phase through stepwise dissolution using HF and an HF–HNO_3_ mixture, respectively. Their results revealed opposite trends in gallium distribution: a higher fraction of gallium was found in the glass phase of fly ash, whereas it predominantly accumulated in the mullite–quartz phase of the bottom ash. They also analyzed boiler slag and found that gallium accumulated most in the mullite–quartz phase (55%) and to a lesser extent in the glass phase (30%).

Bishop et al. [112] examined coal ash samples from four Canadian coal-fired power stations that use coal from the Western Canada Sedimentary Basin. Their sequential leaching analysis revealed a similar pattern of gallium distribution, with most of the gallium accumulating in the residual fraction: around 85% in fly ash and 95–100% in bottom ash. Differences were noted in the acid-soluble (carbonate) and reducible (organic sulfide) fractions, which typically gathered 5–10% of the element. No gallium was found in the ion-exchangeable or oxidizable fractions, nor in the acid-soluble fraction of the bottom ash.

Karan et al. [129] investigated the speciation of gallium in Indian coal fly ash (from a power plant in Talcher) and its various particle fractions. They found that over 95% of the gallium was associated with the residual fraction, with smaller amounts linked to metal oxides, and only trace amounts bound to carbonate or organic/sulfide fractions. No ion-exchangeable forms of gallium were identified. Gallium speciation in coal fly ash from other industrial sources showed some differences [130]. In bituminous coal fly ash (49 ppm Ga) from a thermal power plant in Jharkhand state, 72% of gallium was associated with the mullite–quartz phase, 10% was water-soluble, 9% was organically bound, and 7% was in the ion-exchangeable form, with the remainder in the glass phase. In contrast, lignite fly ash (32 ppm Ga) from a power plant in Southern India contained 36% of gallium in an organic-bound form, 26% in the mullite–quartz phase, 28% in amorphous glass, and the rest in the water-soluble fraction.

Zheng et al. [131] studied fly ash samples collected from the Togtoh Power Plant, which burns coal from the Jungar Coalfield at 1500 °C. They observed a strong positive correlation between gallium concentration and the glass phase, along with a negative correlation with mullite content, indicating a different trend compared to the findings of Feng et al. [114], but consistent with Xu et al.’s data [126].

Gallium was also found to be distributed between the non-magnetic (~45%) and magnetic (~55%) fractions of the ash [126,131]. On the other hand, Dai et al. [116] analyzed fly ash samples from the Jungar Power Plant. They found that the glass phase (40 ppm Ga) made up nearly 55% of the ash, the mullite–corundum–quartz phase (49 ppm Ga), accounted for approximately 43%, while the remaining portion (2%) consisted of the magnetic phase (13 ppm Ga).

### 3.3. Gallium Pre-Enrichment Methods

**Figure 7:**
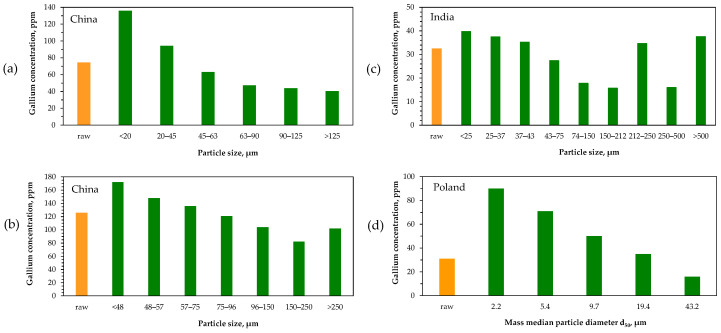
Gallium concentration in particle fractions of industrial coal fly ashes: (**a**) pulverized coal Togtoh Power Plant (1500 °C), China [131], (**b**) circulated fluidized boiler (900 °C), Xinganmeng, China [127], (**c**) pulverized coal power plant, Talcher, India [129], (**d**) hard coal power plant (1480 °C), Poland [132].

Modes of gallium occurrence in coal combustion products indicate that the element tends to accumulate in different phases, particularly in aluminosilicate minerals with non-magnetic properties. Various studies [116,123,126,127,129,130,131,132] have shown that gallium concentrations in fly ash vary with particle size, and a consistent trend is generally apparent across materials from different sources (Figure 7). For instance, in fly ashes generated from the combustion of hard coals in Polish power plants operating at 1450–1600 °C, gallium concentration tends to increase as particle size decreases [123,131]. However, this enrichment pattern does not hold for lignite ashes [123]. Other research has also demonstrated comparable relationships, although mass fractions of particular particle fractions are variable and depend on combustion conditions and coal origin [116,126,127,129,130,131].

Zhou et al. [127] proposed an integrated procedure involving physical separation techniques for the preconcentration of gallium and other critical elements in coal fly ash (126 ppm Ga). They analyzed CFB fly ash with a particle size range from below 48 μm to above 250 μm (Figure 7b), finding that the dominant component was glass (50%). Their analysis identified the main gallium occurrence modes: 77% in residual fractions and 10% carbonate-bound. They determined gallium concentrations in mineral (136 ppm) and glass (117 ppm) phases, as well as in magnetic (77 ppm) and non-magnetic (136 ppm) phases. The magnetic phase, comprising glass, hematite, and mullite, made up 13% of the material, while the non-magnetic phase, consisting of glass and mullite, accounted for 87%. Additionally, they examined gallium distribution in different density fractions, finding the highest concentrations of 171 ppm and 193 ppm Ga in fractions with densities of 2.4–2.6 g/cm^3^ and 2.6–2.8 g/cm^3^, respectively. These results suggest that gallium is enriched in moderate-density fractions, where it is primarily associated with mullite and glass. These fractions can be selectively separated through flotation, while magnetic separation can be employed due to gallium’s accumulation in the non-magnetic phase. Desilication was deemed unsuitable for gallium separation.

A similar procedure was used by Li et al. [148] for the pre-enrichment of valuable elements in fly ash (66 ppm Ga) from a pulverized coal furnace. As with CFB fly ash, the density fraction above 2.4 g/cm^3^ revealed the highest gallium concentration (94 ppm). However, considering different physical separation methods, it was concluded that the optimal approach for gallium concentration is its accumulation in the non-magnetic portion of the material within the particle size fraction below 45 μm.

### 3.4. Gallium Extraction

A promising approach to extracting gallium essentially from coal fly ashes involves various leaching techniques, which have been explored to optimize the recovery of this valuable element from combustion by-products. The presence of aluminosilicate gallium-hosting phases in coal ashes necessitates specialized treatment methods to break the mineral’s bonds and release metal ions during leaching followed by selective separation due to similar chemical properties of gallium and aluminum ions. While most research has concentrated on aluminum recovery from coal fly ash due to its abundant presence, the expertise gained from these efforts has contributed to developing processes for extracting other valuable metals [37,112,133,134,135,136]. However, studies specifically targeting the recovery of gallium remain relatively scarce [37,119,137,138,139,140,141,142,143,144]. It is important also to note that metal extraction efficiency depends on the type of coal ash [145]. The mineral composition of circulating fluidized bed (CFB) fly ash differs from that of pulverized coal (PC) fly ash, primarily due to the lower combustion temperatures in CFB boilers (800–950 °C) compared to PC furnaces (1300–1700 °C), although the origin and type of feed coal also play a significant role [145,146,147,148]. Consequently, CFB fly ash and lignite fly ash have a higher proportion of the amorphous phase and a lower percentage of the magnetic fraction, which can enhance metal reactivity and facilitate easier metal dissolution [145,147].

Chen et al. [133] studied the effect of combustion temperature (400–1300 °C) on phase evolution, particle size, specific surface area, and morphology of high-alumina coal fly ashes produced under laboratory conditions, and their influence on the leachability of valuable elements, including gallium. They observed a gradual increase in particle size and a reduction in surface area, alongside enhanced formation of crystalline phases as the temperature increased. These transformations were accompanied by minor variations in gallium leachability in hydrochloric acid (20% HCl, 95 °C, L/S ratio of 5, 2 h), ranging from 20% to 30%, with the highest leaching efficiency observed for ash produced at a combustion temperature of 800 °C. Notably, the use of an alkaline leachant (NaOH) proved completely ineffective under the same leaching conditions.

Ma et al. [134] proposed a five-step leaching process using 6.3 M HCl and 5 M NaOH solutions at an elevated temperature of 90 °C. This method alternated between acid and alkali treatments in the sequence acid–alkali–acid–alkali–acid, with each stage lasting 2 h. The process achieved a total gallium extraction of approximately 85%, with most gallium being dissolved by the HCl solution, especially during the first acid treatment, which accounted for about 45% of gallium recovery. Although the alkali treatment was much less effective, the alternating conditions facilitated the gradual interruption of Si–O–Al bonds and the removal of SiO_2_, thereby releasing metal ions from the fly ash during acid treatment.

**Figure 8:**
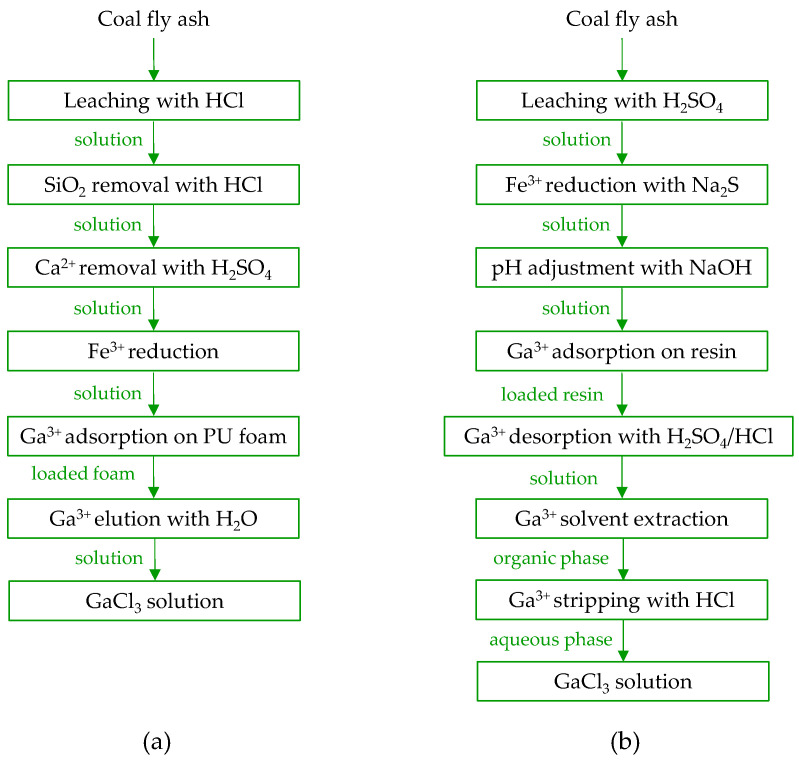
Flowsheets of gallium recovery from coal fly ashes: (**a**) [137], (**b**) [119].

In the 1990s, methods for gallium recovery were developed and patented, encompassing comprehensive stages from acid leaching to the final product [37]. Fang and Gessler [137] explored various leaching parameters, including HCl concentration (1–8 M), liquid-to-solid ratio (2–6), leaching time (2–30 h), temperature (25–65 *°C*), and particle size (<150 mesh to >70 mesh). They addressed impurity removal by methods such as silica hydrolysis with HCl, precipitating Ca^2+^ as sulfate, and reducing Fe^3+^ to Fe^2+^ (method not specified). Additionally, they optimized conditions for gallium extraction on polyurethane foam, examining chloride concentration (3–10 M), sulfuric acid addition (up to 9 M), and temperature (4–55 °C), followed by the final elution of gallium with water. As a result, they developed a comprehensive process for recovering gallium from coal fly ash, producing a gallium chloride solution as the end product (Figure 8a).

Tsuboi et al. [119] established a process for gallium recovery from coal fly ash (Figure 8b) that involves leaching with sulfuric acid H_2_SO_4_, followed by preconcentration of metal ions on a chelating resin with iminodiacetic functional groups and subsequent separation using solvent extraction with tri-n-octylmethylammonium chloride. The leaching step, conducted with 1.5 M H_2_SO_4_, resulted in low gallium ion concentrations (2.8 mg/L) and leaching efficiency (2.4%). However, the adsorption–elution stages significantly increased the gallium concentration to 4000 mg/L. Due to the low selectivity of the leaching and adsorption processes, and the accumulation of vanadium, aluminum, iron, and titanium ions in the eluate, solvent extraction was employed to recover gallium. The final concentration of gallium in a 0.1 M HCl stripping solution reached 3700 mg/L, with a purity of 97%, although iron ions remained the main contaminant.

More recently, unconventional acid leaching methods have been proposed [135,139]. Li et al. [135] reported an energy-efficient leaching process for the preparation of aluminum sulfate with the synergistic extraction of gallium and lithium. They utilized the exothermic effect of diluting concentrated sulfuric acid (to 25–50%), achieving self-heating (100–180 °C) and self-pressure leaching of CFB fly ash at various leaching times (1–5 h) and liquid-to-solid ratios (2–6). The study was conducted on both laboratory and pilot scales, achieving a gallium extraction efficiency of 80–86%. Gallium leaching efficiency increased to nearly 90% after the prior removal of iron from the fly ash via carbothermal reduction magnetic separation. Iron removal by HCl leaching proved to be less effective for gallium recovery, achieving 85% efficiency. This treatment produced a qualified Al_2_(SO_4_)_3_ product from coal fly ash, demonstrating a high-value utilization of the waste material.

Huang et al. [139] compared also the extraction of gallium from coal fly ash using two methods: open wet digestion (120 °C, 3 h) and microwave-assisted digestion (180 °C, 0.5 h, 800–1500 W). They tested mixtures of concentrated hydrochloric acid HCl, hydrofluoric acid HF, and nitric acid HNO_3_. Their results showed that HCl–HNO_3_ and HCl–HF mixtures extracted only about 40% of gallium using open wet digestion, with a slight improvement (by 5–10%) when microwave digestion was applied. In contrast, HNO_3_–HF and HNO_3_–HCl–HF mixtures yielded significantly higher extraction efficiencies of 93% and 83%, respectively, under open wet digestion. For microwave-assisted digestion, the same mixtures achieved 58% and 78% efficiency, which further increased to 97% with extended dissolution time (0.75 h) and higher microwave power (1400 W) when using all three acids. These experiments, although conducted on a laboratory scale, highlight promising directions for optimizing leaching conditions to break down Al–O–Si bonds in aluminosilicate gallium-hosting phases.

Improvement of gallium leaching efficiency can be achieved by employing less aggressive leachants and milder leaching conditions when combined with effective pre-treatment of coal fly ash. Huang et al. [138] explored an alkali roasting method followed by acid leaching, using sodium fluoride NaF, sodium hydroxide NaOH, or sodium carbonate Na_2_CO_3_ as roasting additives under varying parameters (250–900 °C, up to 1 h). The roasted materials were subsequently leached with HNO_3_ solutions (1–5 M) at different temperatures (25–140 °C). They identified NaF as the most effective roasting agent, as it promoted favorable transformations in the mullite host phase and facilitated the conversion of quartz into leachable nepheline, leading to higher gallium recovery compared to direct acid leaching. However, challenges such as acid evaporation and the formation of difficult-to-filter silica gel at higher acid concentrations, which adsorbed gallium ions, resulted in losses. Despite these issues, a maximum gallium extraction efficiency of 94% was achieved with 2 M HNO_3_.

Gao et al. [141] improved gallium extraction from fly ash by roasting the material with Na_2_CO_3_, which disrupted the amorphous phase and converted aluminosilicates, alumina, and silica into more soluble sodium silicate and aluminate. Subsequent leaching resulted in a gallium extraction efficiency of 17% with water, 90% with 3 M HCl, and an approximately 10% higher rate when using 0.5–2 M citric acid HCit solutions. They developed a two-step continuous leaching method, employing 1 M HCl in the first stage and 2 M HCl in the second. This approach yielded 2.6% gallium recovery in the initial stage and 84% in the second, facilitating selective separation from lithium, which leached more efficiently in the weaker acid solution. The formation of silicic acid gel during leaching influenced the selective separation, as density functional theory calculations showed higher adsorption energy of silicic acid for gallium ions compared to lithium ions. This insight suggested that silicic acid gel formation could be strategically used for the selective recovery of these metals.

Gao et al. [143] developed an innovative process for gallium extraction that simultaneously produced mesoporous silica in situ. By calcining fly ash with sodium and/or potassium carbonates, mullite and quartz were converted into nepheline and/or kaliophilite. Subsequent acid leaching (2.5–30% HCl) at room temperature gradually extracted gallium along with aluminum, potassium, sodium, and lithium ions, but left silicon intact. The optimal gallium recovery of 80–83% was achieved using the most concentrated acid solutions within 2 h. Notably, the acid solution also influenced the morphology of the solid residue, generating valuable porous silica with tailored pore size and shape.

The two-stage process of alkali fusion and organic acid leaching was reported by Li et al. [136]. Similar to observations in inorganic acid leaching, the efficiency was enhanced when a roasting stage was included. Among various roasting additives tested (Na_2_CO_3_, NaOH, NaCl, CaO, CaCO_3_, (NH_4_)SO_4_, CH_3_COONH_4_, NH_4_Cl), sodium carbonate showed the best effect. Citric acid emerged as the most effective organic acid for leaching, outperforming tartaric and lactic acids. Through optimization of roasting and leaching parameters (time, temperature, and substance ratios), gallium recovery rates of 80–93% were achieved. This improvement was related to the breakdown of amorphous aluminosilicate and silica phases during alkali fusion, forming nepheline NaAlSiO_4_ and soluble aluminosilicate phases that were easily dissolved by citric acid.

Bioleaching conditions were also tested. Su et al. [149] developed a combined method involving hydrothermal treatment of fly ash using either an alkali (NaOH) or acid (H_2_SO_4_) activator, followed by leaching with *Aspergillus niger*. The initial treatment increased the specific surface area by disrupting the structure of ash particles, thereby enhancing conditions for microbial extraction. *Aspergillus niger* produced organic acids as metabolic byproducts, with concentrations dependent on the medium used for cultivating the culture system. Oxalic acid was the dominant product, but other acids such as acetic, citric, malic, α-ketoglutaric, and succinic acids were also detected. Maximum gallium leaching rates reached 71% for alkali-pretreated ash and 58% for acid-pretreated ash, both surpassing the 39% leaching rate achieved with untreated fly ash, demonstrating the method’s potential application.

Optimal conditions for gallium recovery from coal combustion fly ashes are summarized in Table 5. There is a general lack of information in the literature regarding the recovery of gallium from bottom ash and boiler slag.

### 3.5. Industrial Implementation

**Figure 9:**
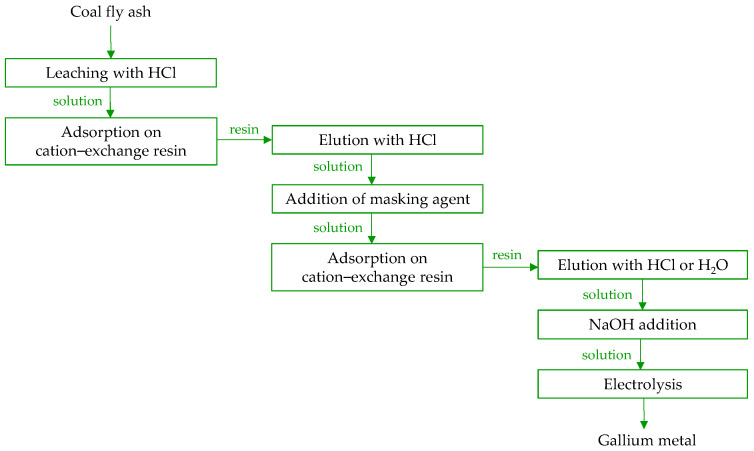
Flow diagram of Shenhua’s patented technology of gallium recovery [152].

Currently, most gallium extraction experiments have been conducted at the laboratory scale, with only a limited number of studies extending to pilot-scale investigations. Nevertheless, recovering metals from power station combustion residues holds significant commercial and environmental interest. Following the discovery of coal with high alumina and gallium contents of the Jungar Coalfield in China, estimated to be 54,000 tons of gallium, the Heidaigou Surface Mine became a focal point for developing technologies to extract these valuable metals from coal combustion CFB fly ash [48]. In 2011, Shenhua Group Zhungeer Energy Corporation Ltd. established the first pilot plant designed to produce gallium and alumina from coal fly ash, with an annual output capacity of approximately 150 tons of gallium and 800,000 tons of Al_2_O_3_ [97,150]. In 2015, Ling [151] informed that Shenhua developed the “one-step acid dissolution method”, a technology for alumina extraction, which enabled the production of 4000 tons of first-grade alumina and 360 kg of 4N-purity gallium. By 2014, a pilot plant had successfully produced alumina with a purity of 99.35%, exceeding the standard for first-grade alumina (98.6%), along with gallium of 99.99% purity. Shenhua’s patented technology [152] outlines a comprehensive process for gallium recovery from crushed circulating fluidized bed fly ash, purified via magnetic separation from iron (Figure 9). The method includes (1) leaching with concentrated HCl at high temperature under pressure (100–200 °C, 0.1–2.5 MPa), (2) adsorption of gallium ions from the acid leachate onto a cation-exchange resin, (3) elution of the resin using diluted HCl, (4) addition of a masking agent (e.g., iron powder, vitamin C) to the elution solution for ferric ion reduction and complexation, (5) passing the treated solution through cation-exchange resin and subsequent elution with water or diluted HCl, (6) adding concentrated NaOH solution to precipitate impurities and prepare the gallium electrolyte, and (7) electrolytic recovery of gallium. Throughout steps (2) to (7), specific temperature conditions ranging from 30 °C to 90 °C need to be maintained. This should ensure the production of gallium metal at 99.9% purity.

In 2013, Lin [153] stated that Chinacoal Pingshuo Group Co. Ltd. had constructed a pilot-scale experimental facility in 2012 to extract silica, aluminum, lithium and gallium from coal fly ash, but no detailed information is available in the literature.

Despite reports of commercial projects in China and other countries working to develop processes for gallium recovery from coal fly ash [154], specific details regarding these initiatives and their current status remain unavailable.

## 4. Gallium in Coal Gangue and Its Combustion Products

### 4.1. Gallium in Coal Gangue

**Figure 10:**
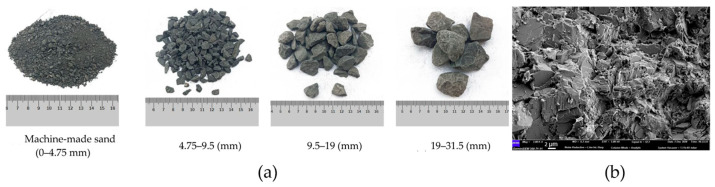
Coal gangue: (**a**) particle fractions, (**b**) SEM image of particle surface [156].

Coal mining generates considerable amounts of waste, particularly in the mining and washing processes [121]. One of the most predominant types of solid waste is coal gangue [155], which typically constitutes about 15–20% of the total raw coal output. Coal gangue is a black-gray shale rock (Figure 10) that is harder than coal and contains a relatively low carbon content (up to 30%). Due to its poor calorific value, high inertness, and limited potential for direct use, coal gangue is often stockpiled in large heaps near mining sites, where it is exposed to weathering.

The chemical composition of coal gangue is primarily dominated by oxides, including up to 70% SiO_2_, 40% Al_2_O_3_, and 15% Fe_2_O_3_. The mineral components of coal gangue include quartz, kaolinite, pyrite, boehmite, mica, as well as traces of heavy metals and potentially valuable elements such as gallium [157,158,159,160,161,162,163,164,165,166,167,168,169]. These factors make coal gangue both a challenging waste material to manage and a potential resource for secondary extraction or industrial applications. The presence of heavy metals, however, raises environmental concerns, particularly in terms of leachate production from weathered long-term storage gangue piles [155].

Table 6 shows gallium concentration and the main gallium-hosting components of some Chinese coal gangues. Metal contents in coal gangues have been identified as higher than in coals (17.6 ppm vs. 6 ppm [157]) and can meet a cut-off grade of 30 ppm to have economic value to gallium recovery. Although there is no industrial implementation of any process, it shows potential if comprehensive utilization of the material is involved (e.g., with aluminum compounds, titania pigments, and silicon-series products) [157].

Gallium often substitutes aluminum in mineral structures due to similar ionic radiuses, thus it can be incorporated into crystal lattices of kaolinite, boehmite, and aluminosilicates (clay minerals) [159,160,161,162,164,165,166,167]. Its association with other minerals like calcite, and sulfides is less common [160,161,163]. Liu et al. [161] calculated the distribution of gallium among minerals in coal gangue showing that almost 85% exist in kaolinite, 7.6% in pyrite, 3.8% in illite, 2.6% in boehmite, 1.1% in muscovite, in which gallium concentrated at levels of 250–340 ppm at a mean gallium concentration in coal gangue of nearly 39 ppm. Sequential leaching procedures confirm speciation of gallium in the waste indicating its predominant occurrence in aluminosilicate residual fraction (88–97%), while carbonate or sulfide fractions host the element in much lower proportions (1–3%) [161,162,163,168,169]. Such distribution mode applies also to different particle size fractions, indicating about 97% of gallium is accumulated within these three forms [160]. Organic matter association is rarely indicated [162,167], while water-soluble or ion-exchangeable forms of gallium in coal gangue practically do not exist.

### 4.2. Gallium Pre-Enrichment Methods

Physical separation methods [121] are commonly used in coal preparation to reduce mineral impurities and enhance the heating value of coal. These methods, which include gravity (density) separation, particle size separation, flotation and magnetic separation, have also been utilized to enrich gallium in coal fly ash (Section 3.3), while their application for the enrichment in coal gangue has been rarely reported.

Zhang et al. [162] investigated various approaches to enriching gallium in coal gangue. They found that gallium concentration increased with particle size, a trend opposite to that observed in coal combustion fly ash (Figure 7). Specifically, the highest gallium concentration (46 ppm) was detected in the largest particles (0.25–0.5 mm), while the lowest concentration (42 ppm) appeared in the finest-grained fraction (<0.075 mm). The sieving method was deemed impractical when compared to the average gallium concentration (43 ppm) in the raw material. Significantly better results were achieved using gravity separation via the conventional float–sink method, employing tetrachloromethane and tribromomethane to prepare separation media with varying specific gravities (2.0–2.6 g/cm^3^). The highest gallium concentration (60 ppm) was recorded in the density fraction above 2.6 g/cm^3^ (enrichment factor of 1.39), whereas no enrichment or depletion occurred in fractions below this density. Unfortunately, the fraction optimal in terms of gallium content had the lowest yield (4.7%). Calcination of coal gangue proved more effective, raising the gallium concentration to 58.6 ppm at 800 °C (enrichment factor of 1.36). Ultimately, Zhang et al. proposed a combined process involving density separation followed by calcination as the most effective method to improve gallium recovery during subsequent acid leaching.

Dai et al. [163] conducted sorting experiments to pre-enrich gallium (and lithium) in coal gangue, focusing on the elements’ modes of occurrence. Mica and clay minerals were identified as the primary carriers of gallium, while gallium showed a negative correlation with iron content. The coal gangue, containing 25 ppm of gallium, was distributed in a single layer using a vibrating feeder on a semi-industrial testing platform. As the coal gangue fell, an XRF sensor detected and analyzed its fluorescence to identify the material with iron content equal to or above a threshold value of 2%. These iron-rich particles were directed into a tailings bin using an electromagnetic hitter, while particles with iron content below the threshold fell naturally into a concentrate bin. The sorting process resulted in a concentrate yield of 68.8%, with a gallium concentration of about 28 ppm, while the gallium concentration in the tailings was approximately 18 ppm. Although this method only slightly enriched gallium in the concentrate, it proved highly effective for lithium upgrading.

Fang et al. [164] proposed flotation as a sorting method for gallium (and lithium). They identified clay minerals as the main gallium carrier minerals in coal gangue (30.5 ppm Ga). Flotation experiments were carried out using a single-cell laboratory flotation machine (80 g/L, 1800 rpm, 0.1 m^3^/h aeration rate, 3 min), employing diesel oil as the collector and sec–octanol as the foaming agent. The results indicated that most of the gallium was concentrated in the tailings (37 ppm), achieving an enrichment factor of 1.21 relative to the original sample, with a recovery rate of gallium-bearing minerals of approximately 66%. Although concentrates contained about 25 ppm Ga, the authors suggested that the tailings could be further dissociated and re-floated, which would reduce grinding time and lower processing costs.

### 4.3. Gallium Extraction

Understanding the gallium occurrence modes in coal gangue is vital for its effective hydrometallurgical extraction. Aluminosilicates as main gallium carriers, exhibit good resistance to water, acids, and alkalis under normal temperature and pressure conditions. Consequently, coal gangue shows low activity and efficiency during leaching. For example, Zhang et al. [162] reported that about 2.5% of gallium was extracted from coal gangue during leaching in 2 M HCl at slightly elevated temperature (60 °C, 4 h), independently of the type of fraction (size, density) selected from the raw material. Direct or grind (in a planetary ball-mill) leaching of coal gangue in ammonium salts (chloride and/or sulfate) solutions or roasting (400 °C) of coal gangue with ammonium salts followed by water leaching were ineffective procedures in terms of gallium dissolution [168]. Therefore, an activation stage in specific conditions is necessary as the initial step in the successful hydrometallurgical recovery of gallium (Table 7).

Calcination is a commonly used method to convert aluminosilicates by breaking down their lattice structure. This occurs due to the transformation of kaolinite into metakaolinite, which results from the loss of structural and interlayer water [166,167]:

Al_2_O_3_∙2SiO_2_∙2H_2_O → Al_2_O_3_∙2SiO_2_ + 2H_2_O      at 450–850 °C (1)

followed by the destruction of silicon–oxygen tetrahedrons:

Al_2_O_3_∙2SiO_2_ → Al_2_O_3_∙(2 − x)SiO_2_ + xSiO_2_      at 850–950 °C (2)

decomposition of metakaolin:

Al_2_O_3_∙(2 − x)SiO_2_ → γ–Al_2_O_3_ + (2 − x)SiO_2_      at 950–1100 °C (3)

and recombination of γ–Al_2_O_3_ and amorphous SiO_2_ into mullite:

3γ − Al_2_O_3_ + 2SiO_2_ → 3Al_2_O_3_∙2SiO_2_         above 1100 °C (4)

**Figure 11:**
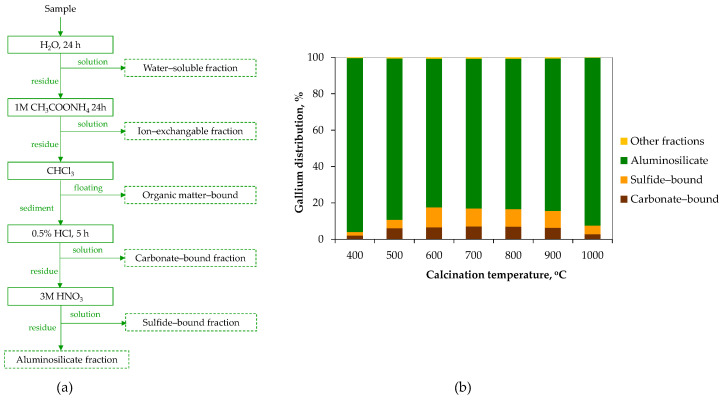
Scheme of sequential leaching procedure (**a**) used for determination of gallium occurrence modes in calcined coal gangue (**b**) [161].

Liu et al. [161] investigated the behavior of gallium during the thermal treatment of coal gangue over a temperature range of 300–1000 °C. They found that calcination triggers a sequence of processes, including the combustion of organic matter, oxidation of pyrite, decomposition of calcite, and dehydroxylation of aluminosilicates (transforming kaolinite into metakaolin), followed by the conversion of metakaolin into mullite. As these mineral phases changed, gallium gradually migrated from the aluminosilicate phase (primarily kaolinite) to iron, potassium, magnesium, and calcium oxides, and then back to aluminosilicates at 1000 °C. By employing a sequential leaching procedure, the researchers tracked gallium transformations and its distribution across various temperatures (Figure 11). They observed a gradual increase in the proportions of gallium in sulfide and carbonate fractions, peaking between 600 °C and 900 °C. This corresponded to reduced shares of gallium bound in the aluminosilicate fraction of 83 ± 1%, compared to 98 ± 1% in the raw material and in the sample calcined at 1000 °C. Other forms of gallium, such as water-soluble, ion-exchangeable, and organic-matter-bound, constituted entirely only a minor fraction (below 0.7%). These phase transformations during calcination can either facilitate or inhibit mineral dissolution, influencing the release of gallium during subsequent leaching processes.

Zhang et al. [162] examined the changes in gallium speciation in coal gangue (43 ppm Ga) induced by calcination at temperatures ranging from 400 °C to 950 °C, employing sequential leaching analysis. At 400 °C, they observed the elimination of carbonate-bound gallium and traces of ion-exchangeable forms, a slight decrease in the proportion of insoluble gallium species, and an approximately tenfold increase in metal oxide-bound gallium portion (to about 13%). Interestingly, the fraction of gallium associated with organic matter increased two- to fourfold within the 400–800 °C temperature range. Calcination was found to reduce the proportion of insoluble gallium forms from 88.5% in the raw coal gangue to 75%, although this proportion increased back to 88.6% at 950 °C. These changes were attributed to the decomposition of calcite and the transformation of kaolinite into mullite. Such modifications in the mineralogical hosting phases of gallium significantly influenced acid leaching efficiency (2 M HCl, 60 °C). Thermal treatment enhanced gallium dissolution from 2.5% for the raw coal gangue to about 54% for the calcination at 400 °C. However, further temperature increase led to a gradual decline in gallium recovery, dropping to below 15% for the calcination at 950 °C. Importantly, the optimal calcination-leaching temperature did not correspond to the calcination temperature yielding the highest gallium enrichment (enrichment factors of 1.06 at 400 °C and 1.36 at 800 °C).

The influence of calcination temperature on gallium extraction (37 ppm Ga in the raw material) was investigated by Shao et al. [166]. They reported a decline in gallium recovery during autoclave leaching with HNO_3_, with recovery rates decreasing from 90% at a calcination temperature of 400 °C to 55–60% for activation temperatures between 500 °C and 800 °C. Gallium recovery from coal gangue calcined at 550 °C generally ranged from 50% to 75% and demonstrated a decreasing trend with increasing leaching temperature (80–170 °C), leaching time (0.5–2 h), and liquid-to-solid ratio (3–6). However, a positive effect was observed with an increase in acid theoretical dosage (0.8–1.07). The duration of calcination (0.5–3 h) had no significant impact on the extraction rate. Notably, the use of HNO_3_ as a leaching agent proved effective for dissolving valuable metals, including gallium, lithium, and aluminum, while leaving iron behind in the solid residue.

A somewhat different behavior of gallium during roasting–leaching treatment was documented by Kang et al. [167]. They investigated coal gangue (22 ppm Ga) activated at temperatures of 400–800 °C and subsequently leached using H_2_SO_4_ (3–7 M) under varying conditions of temperature (70–130 °C), liquid-to-solid ratios (3–20), and leaching times (0.5–2.5 h). An increase in gallium extraction (5 M H_2_SO_4_, 110 °C) was observed as the roasting temperature rose from 400 °C (20% recovery) to 650 °C (90% recovery), followed by a gradual decline in gallium dissolution at higher temperatures. Roasting time (up to 2.5 h) and all leaching parameters had a positive effect on gallium recovery, culminating in up to 95% metal extraction. These high recoveries were attributed to the transformation of the inert crystal structure of kaolinite into a highly reactive form. Additionally, the complete combustion of organic matter, which contained 20% of the total gallium, resulted in a reduction in particle size and a looser particle structure, thereby increasing the reaction surface area and enhanced release of the metal ions.

Wei et al. [165] developed a thermal activation and two-stage selective leaching process to recover gallium alongside lithium from coal gangue. They investigated the effects of three activation additives (mixed in a 1:1 ratio with coal gangue): sodium carbonate Na_2_CO_3_, zinc chloride ZnCl_2_, and ammonium sulfate (NH_4_)_2_SO_4_. Each additive was tested under specific roasting conditions (2 h): 800 °C, 600 °C, and 450 °C, respectively. The roasting process led to the formation of new phases in the coal gangue, altering the distribution of gallium and its leaching behavior in water and HCl. No water-soluble gallium phases were generated, regardless of the additive used. However, gallium could be leached with acid when Na_2_CO_3_ (72% recovery) or NH_4_)_2_SO_4_ (33% recovery) were used, whereas ZnCl_2_ showed no effect on gallium leaching. Due to the significant difference in leaching rates between gallium and lithium, ammonium sulfate was selected as the optimal roasting additive for further process refinement. By optimizing parameters such as the coal gangue-to-ammonium ratio, roasting temperature, and time, a recovery scheme was developed. This involved roasting with ammonium sulfate, followed by water leaching to recover 88% of the lithium and subsequent acid leaching of the solid residue, achieving 90% gallium recovery.

Tian et al. [170] applied an orthogonal experimental design to optimize the conditions for high-temperature acid leaching of gallium from coal gangue. The factors considered included HCl concentration, ignition temperature and duration, as well as leaching temperature and time. Through extensive comparative analysis of various experimental conditions, they determined the optimal parameters for achieving a minimum gallium extraction rate of 95%. The best conditions identified were 6 M HCl, an ignition temperature of 600 °C for 0.5 h, an acid leaching temperature of 100 °C, and a leaching duration of 6 h.

Qin et al. [169] proposed an unconventional approach for treating coal gangue by developing a multistep process. This method involved intercalation with potassium acetate CH_3_COOK, delamination in water under ultrasonication, followed by drying, and roasting with (NH_4_)_2_SO_4_. The intercalation step increased the spacing between kaolinite layers from 0.7 nm to 1.3 nm, indicating the successful insertion of potassium acetate into the mineral interlayers. TEM analysis showed a lamellar curling phenomenon on the particle surfaces, caused by the disruption of interlayer hydrogen bonds and atomic interactions. The intercalation and delamination processes reduced the number of kaolinite layers and particle size, while also increasing the specific surface area available for reaction. Subsequent roasting induced compositional transformations, including the breakdown of aluminum-oxygen octahedra and the volatilization of interlayer hydroxyl groups as water. The roasted material was then leached with water. The pretreatment with delamination improved the gallium leaching rate from 26% to 46%, compared to roasting alone. Speciation analysis of both the raw coal gangue and the roasted product indicated significant changes in gallium distribution. Initially, 95% of the gallium in raw gangue was in residual (insoluble) form, but roasting reduced this fraction by half, converting the remaining gallium into ion-exchangeable (about 30%), carbonate-bound, and organic-bound forms (each around 10%).

### 4.4. Gallium in Coal Gangue Combustion Fly Ash

The use of coal gangue as fuel in some power plants leads to the generation of combustion ash, which presents a potential resource for gallium extraction. Understanding the behavior and distribution of gallium within this ash is critical, as high-temperature combustion processes significantly influence its occurrence and affect recovery strategies. Wu et al. [160] studied the transformation of gallium during the combustion of coal gangue (91 ppm Ga) at temperatures of 600–1000 °C. They found that smaller gangue particles were enriched in gallium, which was predominantly present in residual, sulfide, and metal oxide fractions. Throughout combustion, the gallium content in the products decreased gradually. A minor proportion of gallium transitioned into the gaseous state (16–25%), while the majority accumulated in the ash (75–84%, with gallium concentrations of 135–151 ppm) as the residual phase (about 85%). As combustion temperatures increased, the proportion of active gallium (ion-exchangeable, carbonate-bound, and metal-oxide-bound) increased only slightly in the ash, reaching approximately 9%.

Recovery of gallium from coal gangue combustion ash is seldom discussed in the literature or is collectively treated as coal combustion fly ash. An example has been reported by Hou et al. [141], who investigated the recovery of gallium from circulating fluidized bed fly ash (60 ppm Ga) produced from low-calorific-value coal gangue combusted at 800–950 °C in a power plant. The fly ash contained approximately 37% Al_2_O_3_, 43% SiO_2_, and 5% Fe_2_O_3_. The aluminum was mainly present as amorphous alumina or aluminosilicate, both of which exhibited high chemical solubility under experimental conditions, making the ash suitable for the combined extraction of aluminum and gallium via pressure acid leaching using HNO_3_. The optimal conditions for gallium recovery (77%) were identified as a leaching temperature of 220 °C, an initial acidity of 320 g/L, a liquid-to-solid ratio of 4, and a leaching time of 2 h. Under these conditions, most of the iron remained in the solid oxide residue, effectively separating it from the aluminum. The authors also proposed a process flow chart, including the crystallization of aluminum nitrate, which upon calcination produces a mixture of aluminum and gallium oxides, with nitrogen oxides evolved during the process being recycled for nitric acid regeneration.

## 5. Gallium in Coal Gasification Products

### 5.1. Solid Residues

Coal gasification is a key technology for the clean and efficient utilization of coal [171]. It involves converting coal into gas using atmospheric air or oxygen as a gasifying agent under specific temperature and pressure conditions, either in power plants or directly at the deposit site. This process generates synthesis gas (syngas), which can be used as a fuel or as a feedstock for chemical production. Given the increasing number of gasification installations worldwide [172], it is crucial to explore strategies for managing and utilizing waste from these installations, particularly in terms of using solid residues as secondary sources of valuable metals [173,174].

The solid residues from coal gasification consist primarily of ash and/or slag particles along with unburned carbon [175]. Unlike conventional coal combustion, many coal gasification methods produce minimal fly ash (fine slag [173]), as gasifiers typically operate at temperatures exceeding the ash fusion point. Under these conditions, most of the coal’s mineral matter transforms and melts into slag, which encapsulates non-volatile metals and mineral compounds until it cools. Cooling occurs either in a water bath at the bottom of the gasifier or through natural heat dissipation at the bottom of an entrained bed gasifier.

**Figure 12:**
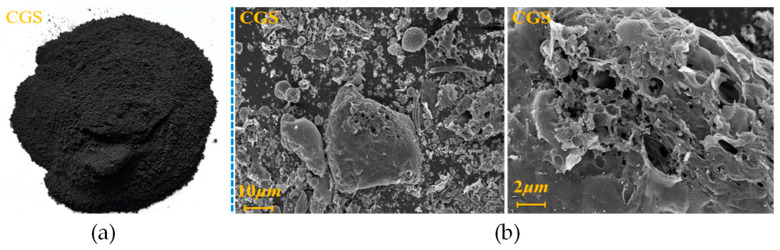
Coal gasification fine slag (**a**) and its SEM images (**b**) [178].

The slag consists of two main components: vitreous frit and residual carbon char (Figure 12). The proportions of these components vary depending on operating conditions, the type of gasifier, and the feedstock, and they can be separated using physical methods. Frit is a black, glassy, silica-based material that is inert, abrasive, and has a low carbon content, forming various shapes from irregular fragments to rod- and needle-like forms. Due to its specific properties and non-hazardous, non-toxic nature, slag can be readily marketed as a by-product for numerous beneficial applications, reducing the need for long-term disposal [176]. Char, the finer component of the gasifier’s solid residues, is composed of unreacted carbon with varying amounts of siliceous ash. It can be recycled back into the gasifier to enhance carbon utilization and has also been employed as a supplemental fuel for pulverized coal combustion [177]. The irregularly shaped char particles have a well-defined pore structure, making them highly suitable for use as an adsorbent and as a precursor for producing activated carbon.

The primary components of coal gasification slag are amorphous inorganic minerals, mainly consisting of SiO_2_, Al_2_O_3_, Fe_2_O_3_, CaO, TiO_2_, K_2_O, MgO, Na_2_O, SO_3_, and P_2_O_5_. The first four components account for 90–95% of the total inorganic matter [177]. The proportions of these minerals vary with particle size: coarse slag generally contains higher proportions of mineral components, whereas fine slag (fly ash) is enriched in unburned carbon, with an average content of 20–30% [173]. Similar to the original gasification slag, the main inorganic components of residual carbon are SiO_2_, Al_2_O_3_, Fe_2_O_3_, and CaCO_3_. The carbon content in this fraction exceeds 95%, and some of it occurs in a partially graphitized form [177].

### 5.2. Gallium in Solid Residues

Minerals contained in the feedstock coal are separated during gasification and leave the gasifier by its bottom slag in high-temperature operation conditions or ash in relatively low-temperature operation conditions [175]. Limited data are available in the literature regarding the gallium content in coal gasification solid products [173,179,180,181,182,183,184,185]. Nevertheless, gallium appears to be primarily enriched in the fly ash compared to the bottom slag or char (Table 8). Moreover, the accumulation of gallium in coal gasification fly ash can reach levels more than ten times higher than those found in the original coal [180,182] and approximately twice as high as in typical coal combustion fly ash [180].

The concentration of gallium in coal gasification wastes depends on the feed coal composition. Arroyo et al. [183] analyzed fly ash samples (containing approximately 200–300 ppm of Ga) from a coal gasification plant in Spain across various operational periods. They observed variations in gallium concentrations ranging from 40 ppm to 60 ppm over weekly and monthly periods, with differences increasing up to 72 ppm over longer sampling intervals (several months or years). This variability was attributed to the use of coal mixtures from newly mined seams, which had higher trace element content compared to other seams. For instance, the gallium content in coal stockpiles built in 2008 was higher (15–22 ppm) than in stockpiles established five or nine years earlier (9–15 ppm).

The mode of gallium occurrence in coal gasification solid wastes remains largely unidentified. However, some insights can be drawn from limited studies on fine slag [180,181]. Phase analysis of the particle size fractions most enriched with gallium revealed an amorphous structure, with quartz identified as a component [180]. Font et al. [181] investigated gallium speciation in fly ash from an IGCC power plant using a sequential leaching procedure developed by the Community Bureau of Reference. The CBR method involves a four-step procedure using the following solutions: 0.1 M acetic acid CH_3_COOH to extract ion-exchangeable or water-soluble species, 0.1 M hydroxylamine chloride NH_2_OH∙HCl to dissolve reducible forms (oxides, hydroxides), 8.8 M hydrogen peroxide H_2_O_2_ to extract, and 1 M ammonium acetate CH_3_COONH_4_ to dissolve oxidizable species (sulfides, organic matter), and finally, a mixture of acids (HNO_3_, HCl, HF) to digest the insoluble residue (aluminosilicates, highly insoluble oxides). The study found that 55% of the gallium was bound to oxides, 20% to the residue phase, and 25% to oxidizable forms (likely sulfides, substituting Zn^2+^ in sphalerite ZnS and/or wurtzite (Zn,Fe)S). The experimental results suggest that gallium is primarily associated with oxides (such as Ga_2_O_3_) and/or embedded within an amorphous matrix structure (substituting for Al^3+^ in aluminosilicates), as well as in sphalerite and/or wurtzite [181,182].

**Figure 13:**
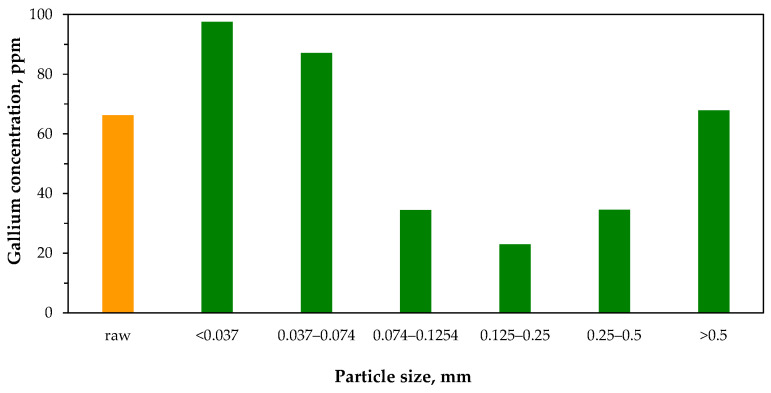
Gallium concentration in particle fractions of industrial coal gasification fine slag [180].

The distribution of gallium among different particle size fractions in coal gasification fly ash appears to differ significantly from that in coal combustion fly ash. Han et al. [180] reported that gallium strongly accumulates in the finest particles (less than 0.074 mm), while particles of intermediate size (0.125–0.25 mm) are notably depleted in gallium (Figure 13). Beneficially, the two-particle fractions with the highest gallium enrichment collectively account for almost 55% of the total mass yield. Regarding enrichment based on density fractions, the highest gallium content (77 ppm) was found in particles with a density greater than 2.0 g/cm^3^, whereas lighter particles contained below-average gallium content.

### 5.3. Gallium Recovery

Data on gallium recovery from coal combustion solid residues are limited [173,182,183], although these wastes have been evaluated for their potential utilization [174,183] or environmental impact [180]. Leachability tests on IGCC fly ash [173] showed very limited gallium dissolution in water and 0.02 M tartaric acid C_4_H_6_O_6_, with solubility depending on sulfuric acid concentration (0.1–1 M) and relatively moderate solubility in sodium hydroxide solutions (0.1–0.5 M). Weak gallium transfer into solution was observed with hydrogen peroxide (oxidizing/reducing agent), oxalic acid C_2_H_2_O_4_ and catechol C_6_H_6_O_2_ (both complexing agents), and lime (alkali agent), while significantly better dissolution was achieved using HCl solutions (0.1–1.0 M) [183].

Notably, the leachability results displayed time- and concentration-dependent trends for different leachants [183]. Divergent leaching efficiencies were observed even for fly ashes with similar gallium contents but from different sampling periods. These scattered results were attributed to variations in element speciation and the possible partial crystallization of the aluminosilicate matrix during fly ash aging. This partial transformation of the amorphous phase can reduce the reactivity of the mineral phase, thereby decreasing the leachable potential of gallium-bearing phases. Nevertheless, the highest leaching efficiency, approximately 59%, was achieved using 0.5 M NaOH after 24 h at 50 °C.

Font et al. [182] investigated gallium recovery from IGCC fly ash (320 ppm Ga) through alkaline leaching with NaOH (0.1–3 M) under various temperatures (25–150 °C), liquid-to-solid ratios (3–10), and durations (6–24 h). Gallium extraction behavior was found to depend on NaOH concentration. The study showed that the process is kinetically controlled when the NaOH molality is below 0.7 M, as the alkalinity is insufficient to dissolve all the gallium. In contrast, for concentrations above 1 M, the process is thermodynamically controlled: although the alkalinity is initially high enough to dissolve gallium, the subsequent precipitation of Na–Al–Si species lowers the NaOH concentration below 0.7 M, reducing gallium extraction yields. The highest gallium leaching rate (99%) was noted at room temperature after 24 h for 1 M NaOH. However, optimal recovery conditions were identified at 0.7–1 M NaOH for 6 h.

Temperature was the most critical factor, with higher temperatures leading to decreased leaching efficiency; at 150 °C, only 10% or less of the gallium was extracted. Recirculating the leachates during the leaching step increased gallium concentrations from 23–38 mg/L to about 200 mg/L after seven cycles, although concentrations then declined. This process also reduced certain impurities, such as zinc and silica, and decreased NaOH consumption.

Gallium precipitation tests showed that 99% of the initial gallium content in the solution could be precipitated by bubbling carbon dioxide CO_2_ until the pH reached 7.4. At a pH of 10.5, significant proportions of impurities (90% or more of Al, Zn, and Ge) were precipitated from the leachates, while 98% of the gallium remained in solution. By re-dissolving this product at pH 0 using HCl and precipitating gallium at pH 3, the Al/Ga ratio in the precipitate was significantly reduced, thereby increasing gallium purity to a level suitable for final purification through electrolysis. Using this method, the final recovery yield for gallium was 132–152 mg Ga/kg of fly ash.

Recently, Pan et al. [179] proposed a method for recovering gallium from coal gasification slag using alkali fusion followed by water leaching. They experimented with a series of roasting additives, including NaOH, KOH, Na_2_O_2_, Na_2_CO_3_, and K_2_CO_2_, at different fusion temperatures (300–1000 °C). They observed opposite trends in subsequent water leaching: gallium dissolution was inhibited as roasting temperatures increased when hydroxides and oxides were used, but improved when carbonates were applied. However, even with sodium compounds as fusion additives, the maximum gallium leaching efficiency did not exceed 50%. Phase analysis of the alkali fusion products and corresponding leaching residues indicated that the formation of insoluble crystalline substances, such as NaAlSiO_4_, was the main factor inhibiting lithium and gallium leaching. Additionally, during the leaching process, sodium silicate Na_2_SiO_3_ present in the alkali fusion products could enhance the dissolution of these insoluble crystals in an alkaline solution, converting them into silicate and aluminate species and releasing gallium. Nevertheless, some of the released gallium could become stabilized within the structure of amorphous substances formed by silicon and aluminum species through isomorphous substitution.

The optimal conditions for gallium recovery from coal gasification fine slags are summarized in Table 9.

## 6. Gallium Separation Techniques

### 6.1. Innovations

Alongside research on gallium extraction from coal waste by-products, efforts are being made to develop methods for separating gallium ions from impurities in leachates. Gallium ion concentrations in these solutions are very low, as the content in the leached solids is in the ppm range. Consequently, classical precipitation methods [144,182] are rarely proposed, with modern techniques like solvent extraction [142,186] and adsorption [187,188,189,190] being more favorable. These methods must address not only the significant concentration differences between gallium(III) ions and impurities but also the chemical similarity of gallium to aluminum and iron(III) ions. Most gallium ion separation studies use synthetic solutions rather than real leachates, making it essential to develop techniques tailored to these complex mixtures, which contain numerous coexisting metal ions. The literature provides only a few examples of such specialized approaches, with some data summarized in Table 10.

**Figure 14:**
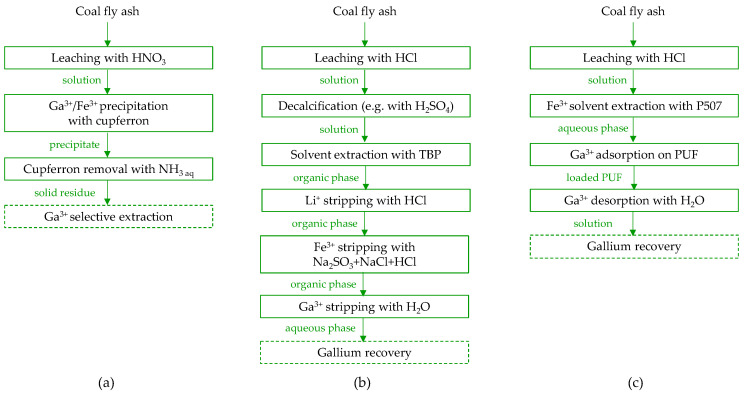
Flow diagrams for gallium recovery from coal fly ash leachates: (**a**) precipitation with cupferron [144], (**b**) solvent extraction [142], (**c**) adsorption on PU foam [188].

Shi et al. [144] proposed a traditional precipitation method using an unconventional precipitant, cupferron C_6_H_9_N_3_O_2_, for the selective separation of gallium and iron from aluminum in solutions. They tested a simulated mixture containing the primary components (Ga^3+^, Al^3+^, Fe^3+^) representative of an HNO_3_ leachate (pH 0–1) from coal gangue [166] and fly ash [141]. By optimizing precipitation parameters such as cupferron dosage, reaction time, and pH, a gallium-to-aluminum separation ratio of 2100 was achieved, with 99% gallium recovery from the solution. The resulting precipitate consisted of gallium and iron chelate complexes with cupferron. The post-precipitation solution contained 67 ppm Ga^3+^, 51 ppm Fe^3+^, and 33 g/L Al^3+^. Based on these results, a complete gallium recovery scheme was proposed, involving stages of HNO_3_ leaching, selective Ga–Fe precipitation using cupferron, leaching of the complex with ammonia for cupferron regeneration, and subsequent iron extraction, leaving gallium as the final product (Figure 14a).

Zhao et al. [186] developed a solvent extraction procedure for gallium recovery from sulfuric acid-based leach liquor. The process involved purifying the leachate using the P507 extractant to remove major impurities, Fe^3+^ and Ti^4+^, with only 1.2% of Ga^3+^ lost. Gallium was then extracted from the aqueous phase in a single step using Cyanex 272 and subsequently stripped with HCl. After scrubbing with H_2_SO_4_, the extractant was regenerated to reuse in the closed-loop system. The method achieved effective separation, with separation factors of 145 for Fe/Ga and 40 for Ga/Al. Additionally, the study discussed the mechanism of gallium extraction and the formation of gallium species within the organic phase.

More recently, Cui et al. [142] developed a solvent extraction process for recovering gallium (100 ppm) and lithium (248 ppm) from simulated hydrochloric acid leachate derived from coal fly ash. By thoroughly examining the extraction and stripping stages, they proposed a flowsheet for the synergistic separation and recovery of aluminum, lithium, iron, and gallium from decalcified acidic chloride solutions (Figure 14b). The process involved a single-step extraction using tributyl phosphate TBP with co-extraction of lithium (87%), iron (98%), and gallium (almost 100%) ions:

Li^+^ _A_ + FeCl_4_^−^ _A_ + 2TBP _O_ ⇄ [Li(TBP)_2_][FeCl_4_] _O_ (5)

Li^+^ _A_ + GaCl_4_^−^ _A_ + 2TBP _O_ ⇄ [Li(TBP)_2_][GaCl_4_] _O_ (6)

followed by three stripping stages: (I) lithium recovery from the Fe–Li–Ga loaded organic phase using 6 M HCl, (II) iron recovery from the Fe–Ga phase with a sodium salt mixture (sodium sulfite and chloride) in HCl, and (III) gallium recovery from the Ga-loaded phase with water, achieving a 96% gallium recovery rate and producing an aqueous solution containing 94 mg/L Ga^3+^. After six extraction–stripping cycles, gallium concentration increased to 514 mg/L, resulting in a total recovery of 82%. The study also discussed the mechanisms of metal ion extraction within the TBP extractant.

Li et al. [187] investigated the direct adsorption of gallium from feed solutions in a pre-desilication soda–lime sintering process. They used actual process liquid from alumina extraction of high-alumina coal fly ash, which contained 75–80% of gallium leached from fly ash. The study employed HF 528 acrylonitrile–divinyl benzene resin functionalized with amidoxime groups capable of chelating gallium species. Activated with NaOH, the resin achieved a saturated adsorption capacity of approximately 2.8 mg Ga/g within 24 h. For the desorption stage, an alkaline sodium sulfide solution (NaOH and Na_2_S) was used. Gallium elution increased from 600 mg/L to 2400 mg/L as Na_2_S concentration rose from 1 M to 2 M. Column adsorption experiments demonstrated the resin’s stability over five cycles.

Zhao et al. [188] conducted experiments using synthetic solutions to develop a process for recovering gallium from real acid chloride solutions (Figure 14c). Following solution purification via solvent extraction with P507, polyurethane foam PUF was used for the selective recovery of gallium, effectively leaving aluminum, calcium, and manganese ions in the solution. The loaded PUF demonstrated efficient gallium elution using water, and the system showed stable performance over 10 adsorption–desorption cycles, maintaining PUF capacities of approximately 16 mg Ga^3+^/g.

The latest advancements in selective adsorption of gallium from acid leaching of coal fly ash have led to the development of innovative adsorbents. Zhang et al. [189] synthesized a graphene oxide-based Ga(III) imprinted polymer GO–PAA using an adsorption method combined with surface ion imprinting technology. This sorbent exhibited a maximum capacity of approximately 220 mg Ga^3+^/g within 4 h in acid chloride solution and showed high selectivity for gallium over competing impurities, with selectivity coefficients of 10 for aluminum, 3.4 for ferric, 18.5 for magnesium, and 55.5 for calcium ions. The reported saturation adsorption capacity was the highest recorded in the literature as of 2024, attributed to the use of graphene oxide as a carrier. The imprinted adsorbent also demonstrated excellent regeneration performance, retaining 86% of its initial capacity after five adsorption–regeneration cycles.

Zhang et al. [190] developed a functionalized Ti_3_C_2_T_x-_based MXene composite aerogel (MPHG–40) for gallium ion adsorption from acid leachate. The aerogel demonstrated high selectivity for Ga^3+^ over copper, zinc, ferric, and aluminum ions in simulated solutions. The maximum adsorption capacity reached nearly 133 mg Ga^3+^/g at pH 3 within 6 h in an acidic nitrate solution, and the performance remained stable with minimal decline after five adsorption–regeneration cycles.

### 6.2. Coal By-Products as Gallium Adsorbent Precursors

Utilizing waste materials for metal recovery offers significant practical benefits, including reducing stockpiled waste. Fly ashes from coal combustion or gasification have proven to be effective precursors for synthesizing mesoporous silica-based adsorbents for gallium ion removal. Li et al. [191] described a hydrothermal method to prepare an adsorbent from coal combustion fly ash leaching residue, creating high-quality mesoporous silica modified with CTAB, which efficiently recovered gallium from synthetic acid chloride solutions (30–400 mg/L Ga^3+^), achieving 80–85% adsorption efficiency over five cycles with desorption using 0.1 M HCl. In turn, Yang et al. [192] used coal gasification coarse slag to generate mesoporous silica via acid leaching, increasing the specific surface area 40-fold compared to untreated slag. Under optimal conditions, the material exhibited 99% gallium adsorption efficiency from an alkaline solution (40 mg/L Ga^3+^), while untreated slag only achieved 3%. These studies highlight the potential of coal by-products for sustainable gallium extraction and resource recovery.

## 7. Materials and Methods

**Figure 15:**
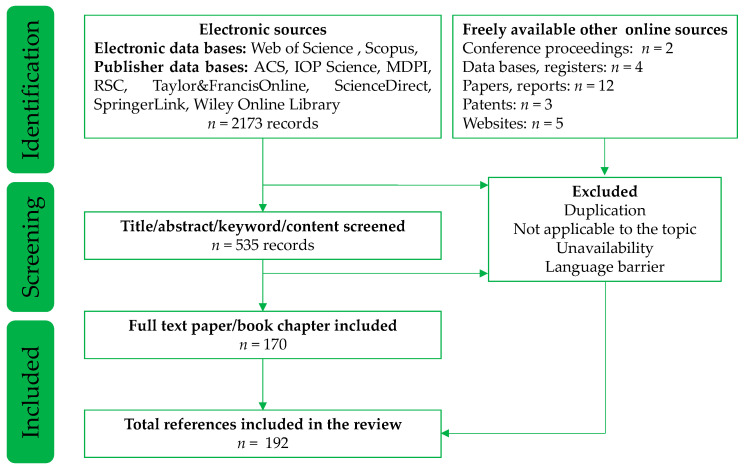
Flow diagram for systematic review research.

The literature search was conducted over the period of September–13 November 2024 focusing on keywords, particularly “gallium”, “coal”, “coal gangue”, “coal fly ash”, “coal gasification ash”, “recovery”, and “leaching”, among others. This systematic literature review is based on 192 references, including scientific publications, books and freely available online information and data, according to PRISMA 2020 guidelines [193] (Figure 15).

Two primary databases were explored: Web of Science and Scopus, as well as databases from specific publishers (ACS—American Chemical Society, IOP Science, MDPI, Royal Society of Chemistry, Taylor&FrancisOnline, ScienceDirect, SpringerLink, Wiley Online Library). The search also included freely available online papers, statistical base data and institutional sources to find detailed information (e.g., gallium prices, production statistics, etc.). Research papers, reviews and books were identified by assessing both the abstracts and contents. Comprehensive coverage of the relevant literature was achieved through cross-referencing citations and reviewing any attached supplementary materials. Attention was paid to the methodological approaches, crucial outcomes, and relevance of the recent studies to the current research context providing a main basis for further analysis and discussion. However, it should be noted that English-language literature does not fully cover the available data on the occurrence and recovery of gallium from coal and its waste products, as this topic has also been intensively explored in Chinese-language publications. These papers were not included in this review due to language barriers and accessibility limitations, which prevented a thorough examination of their content beyond the English abstracts.

## 8. Conclusions

Coal and coal wastes can be alternative sources of valuable metals, including gallium. While gallium-rich coal deposits are primarily concentrated in China, coal combustion and gasification products hold higher potential due to gallium’s tendency to accumulate in these materials. Direct gallium leaching from coal, bottom ash, or coarse slags has not yet been tested. However, coal gangue and fly ashes represent more viable secondary gallium resources due to their simpler composition and higher gallium contents compared to raw coal. Gallium in coal and coal-based materials predominantly exists in inert aluminosilicate phases, which requires a proper pretreatment to break the Si–O–Al bonds, enabling over 90% gallium leaching from coal combustion fly ashes or coal gangue under optimal conditions. Coal gasification produces fly ash with the highest gallium concentration, but recovery rates are reduced due to differences in the proportions of gallium-hosting phases and possible crystallization of the amorphous phase during ash aging. Despite coal gasification generating significantly less fly ash than combustion, the development of industrial plants worldwide should also focus on this material as a potential source of gallium.

Developing selective recovery methods from leachates is challenging due to the very low gallium concentrations (much below 0.5 g/L), accompanied by high amounts of impurities, mainly aluminum, iron, calcium, magnesium, and sodium ions, which have similar chemical properties to gallium ions. Therefore, effective closed-loop solvent extraction methods and advanced sorbents specifically designed for selective gallium recovery must be further developed. Methods for obtaining high-purity gallium products are still under investigation, and this aspect of gallium recovery needs further clarification, although gallium electrolysis is most often suggested.

Currently, gallium extraction experiments are being conducted in laboratories, but they have only gained significant attention in recent years. The lack of pilot-scale investigations presents limitations for common commercial production, though this is expected to be addressed in the future. Integrating gallium recovery with the extraction of aluminum, silicon, and potentially other metals such as rare earth elements could offer a feasible way to reduce production costs. Leaching and other chemical treatments can generate some quantities of hazardous liquid waste containing dissolved heavy metals and other pollutants. This will also require appropriate waste treatment to mitigate the risks of contamination to surrounding ecosystems.

Advances in pretreatment, extraction, and separation processes, along with environmentally friendly waste treatment methods, will undoubtedly enhance gallium production from coal by-products, contributing to a more widespread and sustainable global supply of gallium, but also other valuable elements. Such a complete approach is essential for fully assessing the economic viability of hydrometallurgical processing of coal-based waste materials.

## Figures and Tables

**Table 1:** Gallium concentration in natural sources.

Source	Concentration Range, ppm	Mean Concentration, ppm	Ref.
Continental crust	15–18	16	[32]
upper	14–18	17.5	[32]
middle	17.5–18	17.5	[32]
lower	13–19	13	[32]
Bauxite ore	5–812	52	[8]
karstic (carbonate)	<10–180	58	[30,31]
lateritic (aluminosilicate)	<8–146	57	[30,31]
Zinc sulfide ore	up to 2500–3120 ^1^	no detailed data	[8,28,29]
MVT ^2^	32–56	42	[27]
SHMS ^2^	5–22	11	[27]
VHMS ^2^	1–35	19	[27]
VEIN ^2^	10–19	14	[27]
HTHR ^2^	1.8–5.3	3.1	[27]

^1^ The highest known gallium contents in sphalerite of 2.05–3.7 wt% were found in sulfide nodules in the Qingzhen chondrite (China) [33]. ^2^ Sphalerite deposit types: MVT—Mississippi Valley-type, SHMS—sediment-hosted massive sulfide, VHMS—volcanic-hosted massive sulfide, VEIN—Vein-type (highly heterogeneous), HTHR—high-temperature hydrothermal replacement; geometric mean concentrations [27].

**Table 3:** Modes of gallium occurrence in some world’s coals.

Region	Main Gallium-Bearing Matter; Other	Ref.
Bulgaria		
Pernik Basin	organic matter; authigenic minerals	[52]
China		
Datong Coalfield/Yanzishan Mine	inorganic–organic affinity (organic sulfur)	[100]
Guizhou Coalfield/no details	inorganic–organic affinity	[53]
Heshan Coalfield/Shicun and Heliluoshan Mines	organic–inorganic affinity (sulfate sulfur)	[54]
Jincheng Coalfield/Sihe Mine	kaolinite, diaspore	[98]
Jungar Coalfield/Heidaigou Mine	kaolinite, boehmite; humic acid (adsorbed)	[43,55]
Jungar Coalfield/Haerwusu Mine	kaolinite, boehmite	[98]
Jungar Coalfield/Haerwusu Mine	organic matter (collodetrinite, collotelinite), kaolinite	[95]
Ningwu Coalfield/no details	silicates (kaolinite, boehmite); organic matter, sulfide	[45]
Ningwu Coalfield/Anjialing Mine	silicates, sulfides; water-soluble, organic	[101]
Ningwu Coalfield/Laoyaogou Mine	aluminosilicates; Ti-bearing minerals	[57]
Ningwu Coalfield/Nangou Mine	organic matter; kaolinite	[102]
South Qinling Orogenic Belt	sphalerite, diaspore, arsenides/organic affinity, clay minerals	[46]
Xian’an Coalfield/Wanfu Mine	kaolinite, illite/smectite, chlorite	[103]
Iran		
Shemshak Group coal	inorganic	[60]
Kazakhstan		
Karaganda Basin	sulfur-related	[62]
Turkey		
Sorgun Basin	inorganic materials	[73]
USA		
Indiana/Springfield and Danville Seams	organic biomass, pyrite	[78]

**Table 4:** Gallium concentration (ppm) in coal and coal ashes.

Country	Coal	Ash	Fly Ash	Bottom Ash	Ref.
World	5.5/6.0 *	29/36 *	–	–	[50]
Canada	–	–	27.5	12.1	[112]
China	44.8	81.8	–	–	[43]
China	2.5–24.6	12.2–57.9	–	–	[53]
China	98.8 ± 25.6	–	287.6 ± 84.6	195.6 ± 58.4	[113]
China	28	–	133	36	[114]
China	22.8	–	68.5	31.1	[115]
China	24.3	–	44.4	43.0	[116]
Greece	22.8	–	68.5	31.1	[117]
Greece	–	–	21–39	–	[118]
India	–	–	27/36 *	16/21 *	[111]
Japan	–	–	230	–	[119]
Norway	0.1–0.2	1–12	–	–	[120]
Poland	13.3 ± 5.5	85.5	–	–	[121]
Poland	–	–	43–53/58–82 *	–	[122]
Poland	2.2/2.3 *	–	27–35/26–35 *	–	[123]
Poland	–	48.9/27.2 *	–	–	[124]
Russia	2.0	2.5	–	–	[65,80]
Russia	5.7; 6.2	46.4; 53.3	–	–	[82]
Russia	–	23.5–43.8	–	–	[68]
Turkey	6.6 ± 0.2	13 ± 0.4	13 ± 1.3	11 ± 0.4	[71]
Turkey	5.0–8.3	–	14–19	12–21	[72]
Turkey	6.3–10	–	16–22	9.5–16	[125]
USA	2–9	99.8–215	–	–	[81]

* Mean gallium contents in coals or ashes of brown B (lignite) and hard H (bituminous) coals (B/H).

**Table 5:** Optimal conditions of gallium leaching from coal combustion fly ash.

Coal Fly Ash: Type; Ga Content	Pretreatment Stage	Leaching Conditions	Gallium Extraction	Ref.
no data; 104 ppm	–	2 M HCl, 20 °C, L/S 6, 2 h	77%	[137]
CFB; 118 ppm	–	alternate multistep leaching: acid–alkali–acid–alkali–acid 6.3 M HCl, 5 M NaOH, 90 °C, L/S 5, 2 h	85%	[134]
CFB; 188 ppm	–	40% H_2_SO_4_, 140 °C, L/S 3, 2 h	88%	[135]
no data	alkali roasting: NaF/ash 0.75:1, 800 °C, 0.17 h	2 M HNO_3_, 120 °C, L/S 12, 1 h	94%	[138]
no data	–	HNO_3_ + HF (5:1), 120 °C, L/S 12, 3 h	93%	[139]
no data	–	HNO_3_ + HF + HCl (5:1:5), 180 °C, L/S 12, 0.75 h, 1400 W	97%	[139]
CFB, 350 ppm	alkali roasting Na_2_CO_3_/ash 1:1, 800 °C, 2 h	two-step acid leaching: 1 M HCl–2 M HCl, no details	87%	[141]
no data; 68 ppm	alkali roasting Na_2_CO_3_/K_2_CO_3_ ash, 900 °C, 2 h	30% HCl, 25 °C, L/S 5, 2 h	83%	[143]
PC; 98 ppm	alkali roasting Na_2_CO_3_/ash 1:1, 875 °C, 1.5 h	0.4 M HCit, 50 °C, L/S 125, 1 h	94%	[136]
no data; 62 ppm	NaOH hydrothermal treatment: 150 °C, 24 h	*Aspergillus niger*, 30 °C, 8 days	71%	[149]

**Table 6:** Gallium occurrence in Chinese coal gangues.

Location	Concentration, ppm	Bearing Minerals	Ref.
Huanglong Coalfield/Yuanzigou	1–30	no data	[157]
Inner Mongolia/Xing’an League	85–95	calcite, aluminosilicate	[160]
Inner Mongolia/preparation plant	43	kaolinite	[162]
Jungar Coalfield/no data	89	boehmite, kaolinite	[159]
Jungar Coalfield/Heidaigou Mine	15	aluminosilicate (residual)	[168]
Shanxi Province/Antaibao Mine	25	silicate, sulfide	[163]
Shanxi Province/Jincheng seam	22	silicate, aluminosilicate	[167]
Shanxi Province/Pingshuo Mine	20–30	kaolinite, clay minerals	[164,165]
Shanxi Province/Shuozhou	37–39	kaolinite, pyrite	[161,166]
Weibei Coalfield/Sanghuping Mine	3–50	no data	[157]
Xishan Coalfield/Duerping Mine	37–43	clay minerals	[158]

**Table 7:** Optimal conditions of gallium recovery from coal gangue (gallium concentration in the feedstock is shown in Table 6, according to the references).

Pretreatment Stage	Leaching Conditions	Gallium Extraction	Ref.
One-step pretreatment			
Calcination: 400 °C, 4 h	2 M HCl, 60 °C, L/S 10, 4 h	54%	[162]
Calcination: 400 °C, 2 h	HNO_3_ (TD * 1:1), 170 °C, L/S 5, 2 h	90%	[166]
Roasting: 650 °C, 2 h	6 M H_2_SO_4_, 120 °C, L/S 10, 2 h	95%	[167]
Roasting: (NH_4_)_2_SO_4_/CG (2.5:1), 450 °C, 2 h	2 M HCl, 90 °C, L/S 10, 3 h	90%	[165]
Roasting: Na_2_CO_3_/CG (1:1), 800 °C, 2 h	2 M HCl, 90 °C, L/S 10, 3 h	72%	[165]
Multi-step pretreatment			
I. Intercalation:CH_3_COOK, 25 °C, 28 h II. Delamination: H_2_O, 0.5 h, ultrasound; drying III. Roasting: (NH_4_)_2_SO_4_, 380 °C, 1 h	H_2_O, 70 °C, S/L 1:80, 1 h, stirring	46%	[169]

* TD—theoretical dosage of HNO_3_ related to total metal (Al, Fe, Ca, Mg, K, Na) content in coal gangue.

**Table 8:** Gallium occurrence in coal gasification solid products.

Source	Concentration, ppm	Ref.
China/Slag	38.8	[179]
China/Fine slag	66.2	[180]
Spain/IGCC * Fly ash	186–320	[173,181,182]
Spain/IGCC * Fly ash	212–299	[183]
United Kingdom/Char	14	[184]

* IGCC—Integrated Gasification Combined Cycle.

**Table 9:** Optimal conditions of gallium recovery from coal gasification fly ashes CGFA (gallium concentration in the feedstock is shown in Table 8, according to the references).

Pretreatment Stage	Leaching Conditions	Gallium Extraction	Ref.
–	1 M HCl, 50 °C, L/S 5, 6 h	41%	[183]
–	0.5 M HCl, 50 °C, L/S 5, 2 h	36%	[183]
–	0.5 M H_2_SO_4_, 50 °C, L/S 5, 24 h	66%	[183]
–	0.1 M H_2_SO_4_, 50 °C, L/S 5, 6 h	20%	[183]
–	1 M NaOH, 25 °C, L/S 5, 6 h	86%	[182]
–	1 M NaOH, 25 °C, L/S 10, 24 h	99%	[182]
Alkali fusion: NaOH/CGFA (1:1), 300 °C	H_2_O, 50 °C, L/S 5, 2 h	48%	[179]
Alkali fusion: KOH/CGFA (1:1), 300 °C	H_2_O, 50 °C, L/S 5, 2 h	35%	[179]
Alkali fusion: Na_2_O_2_/CGFA (1:1), 300 °C	H_2_O, 50 °C, L/S 5, 2 h	52%	[179]
Alkali fusion: Na_2_CO_3_/CGFA (1:1), 1000 °C	H_2_O, 50 °C, L/S 5, 2 h	49%	[179]
Alkali fusion: K_2_CO_3_/CGFA (1:1), 1000 °C	H_2_O, 50 °C, L/S 5, 2 h	39%	[179]

**Table 10:** Optimal conditions of gallium separation from leachates (L) and simulated solutions (S).

Solution Type	Ga:Al:Fe Ion Concentration, g/L	Separation Steps	Gallium Recovery	Ref.
Precipitation				
S, acid, nitrate	0.07:34:0.5	25 °C, cupferron (TD 4), pH 0.9, 10 min	99%	[144]
Solvent Extraction				
L, acid, sufate	0.1:42:8.4	Pretreatment: 1.5 M P507 Extraction: 0.5 M Cyanex 272, A/O 2, pH 2.5, 0.5 h Stripping: 2 M HCl, A/O 2, 0.5 h	82%	[186]
S, acid, chloride	0.1:51:4.5	Extraction: TBP, A/O 1, 10 min Stripping: I—Li^+^; II—Fe^3+^ Stripping: III—H_2_O, A/O 1, 0.5 h	99%	[142]
Adsorption				
L, alkaline	0.05:39:0	Adsorption: HF 528 resin Elution: 2 M NaOH + 1.5 M Na_2_S	no data	[187]
L, acid, chloride	0.1:25:3.4	Pretreatment: 1 M P507, pH 0 Adsorption: PUF Desorption: H_2_O	99%	[188]
S, acid, chloride	0.2:?:?	Adsorption: GO–PAA, pH 3 Desorption: 1 M HCl	86%	[189]
S, acid, nitrate	0.05:?:?	Adsorption: MPHG–40, pH 3 Desorption: 1 M HCl	no data	[189]

## Data Availability

No new data were created.
